# A large-scale dataset of patient summaries for retrieval-based clinical decision support systems

**DOI:** 10.1038/s41597-023-02814-8

**Published:** 2023-12-18

**Authors:** Zhengyun Zhao, Qiao Jin, Fangyuan Chen, Tuorui Peng, Sheng Yu

**Affiliations:** 1 Center for Statistical Science, Tsinghua University, Beijing, 100084 China; 2 School of Medicine, Tsinghua University, Beijing, 100084 China; 3 Department of Physics, Tsinghua University, Beijing, 100084 China

**Keywords:** Databases, Health care

## Abstract

Retrieval-based Clinical Decision Support (ReCDS) can aid clinical workflow by providing relevant literature and similar patients for a given patient. However, the development of ReCDS systems has been severely obstructed by the lack of diverse patient collections and publicly available large-scale patient-level annotation datasets. In this paper, we collect a novel dataset of patient summaries and relations called PMC-Patients to benchmark two ReCDS tasks: Patient-to-Article Retrieval (ReCDS-PAR) and Patient-to-Patient Retrieval (ReCDS-PPR). Specifically, we extract patient summaries from PubMed Central articles using simple heuristics and utilize the PubMed citation graph to define patient-article relevance and patient-patient similarity. PMC-Patients contains 167k patient summaries with 3.1 M patient-article relevance annotations and 293k patient-patient similarity annotations, which is the largest-scale resource for ReCDS and also one of the largest patient collections. Human evaluation and analysis show that PMC-Patients is a diverse dataset with high-quality annotations. We also implement and evaluate several ReCDS systems on the PMC-Patients benchmarks to show its challenges and conduct several case studies to show the clinical utility of PMC-Patients.

## Background & Summary

Clinicians often rely on Evidence-Based Medicine (EBM) to combine clinical experience with high-quality scientific research to make decisions for patients^1^. However, finding relevant research can be challenging^2^. To address this issue, there has been increasing research interest in utilizing Natural Language Processing (NLP) and Information Retrieval (IR) techniques to retrieve relevant articles or similar patients for assisting patient management^3–7^. In this article, we introduce the term “Retrieval-based Clinical Decision Support” (ReCDS) to describe these tasks. ReCDS can provide clinical assistance for a given patient by retrieving and analyzing relevant articles or similar patients to determine the most likely diagnosis and the most effective treatment plan.

ReCDS with relevant articles is grounded in EBM. Therefore, the majority of ReCDS studies have focused on retrieving relevant research articles^8–10^, which are primarily facilitated by the Clinical Decision Support (CDS) Track^3,11,12^ held annually from 2014 to 2016 at the Text REtrieval Conference (TREC). Each year, the TREC CDS Track releases 30 “medical case narratives” and participants are asked to return relevant PubMed Central (PMC) articles for each patient. Although sufficient patient-article relevance can be annotated under the TREC pooling evaluation setting^13^, the size and diversity of the test patient set in TREC CDS are limited. Consequently, the generalizability of system performance to uncovered medical conditions may be constrained.

ReCDS with similar patients, on the other hand, is still under-explored. Retrieving the medical records of similar patients can provide valuable guidance, especially for patients with uncommon conditions such as rare diseases that lack clinical consensus. Nevertheless, there are various challenges in conducting this type of research. Unlike scientific articles, there is currently no publicly available collection of “reference patients” to retrieve from. Moreover, defining “patient similarity” is non-trivial^14^ and large-scale annotation is prohibitively expensive. As a result, there are only a few studies on similar patient retrieval^15,16^, all of which use private datasets and annotations.

The aforementioned issues make it clear that a standardized benchmark for evaluating ReCDS systems is greatly needed. Ideally, such a benchmark should contain: (1) a diverse set of patient summaries, which serve as both the query patient set and the reference patient collection; (2) abundant annotations of relevant articles and similar patients. Due to privacy concerns, only a few clinical note datasets from Electronic Health Records (EHRs) are publicly available. One notable large-scale public EHR dataset is MIMIC^17–20^. However, it only contains ICU patients without any relational annotations, making it unsuitable for evaluating ReCDS systems.

**Fig. 1:**
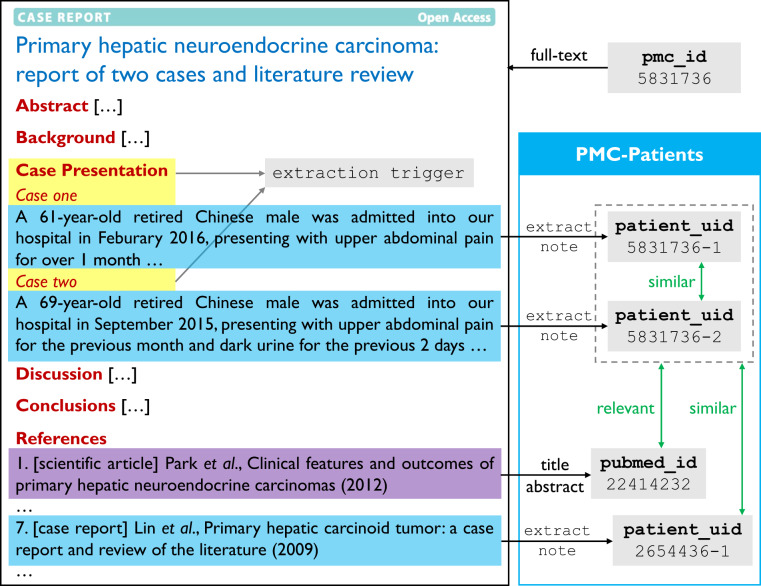
Overview of the PMC-Patients dataset architecture. Patient summaries are extracted by identifying certain sections in PMC articles. The cited articles and patients are considered relevant and similar, respectively. Patients from the same report are also considered similar.

In this article, we aim to benchmark the ReCDS task with PMC-Patients, a novel dataset collected from the case reports in PMC and the citation graph of PubMed. Case reports denote a class of medical publication that typically consists of: (1) a case summary that describes the patient’s whole admission; (2) a literature review that discusses similar cases and relevant articles, which are recorded in the citation graph. To build PMC-Patients, we first extract patient summaries from case reports published in PMC using simple heuristics. For these patient summaries, we then annotate relevant articles and similar patients using the PubMed citation graph. Figure 1 demonstrates the dataset collection via an example. PMC-Patients is one of the largest patient summary collections, with the largest scale of relation annotations. Besides, the patients in our dataset show a higher level of diversity in terms of patient characteristics than existing patient collections. Our manual evaluation shows that both patient summaries and relation annotations in PMC-Patients are of high quality.

**Fig. 2:**
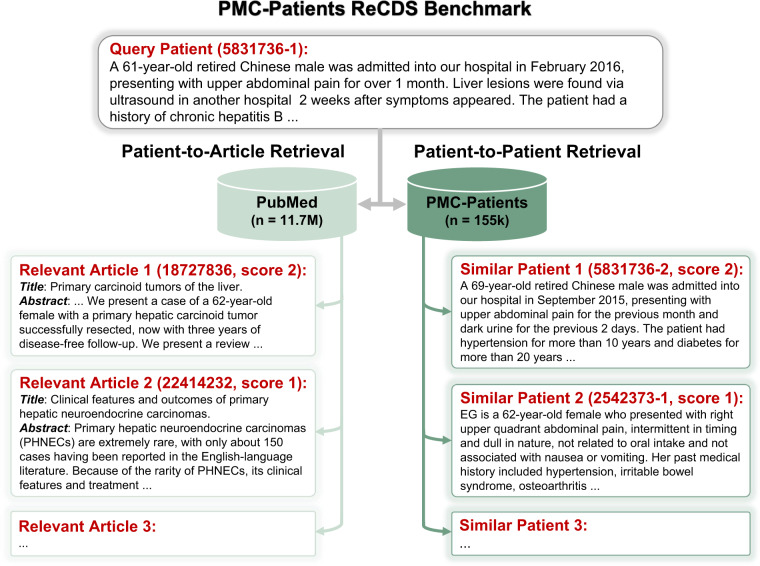
Overview of the PMC-Patients ReCDS benchmark. Given a query patient, there are two tasks: 1. Patient-to-article retrieval requires returning relevant articles from PubMed; 2. Patient-to-patient retrieval requires returning similar patients from PMC-Patients.

Based on PMC-Patients, we formally define two ReCDS tasks: Patient-to-Article Retrieval (ReCDS-PAR) and Patient-to-Patient Retrieval (ReCDS-PPR), which are illustrated by an example in Fig. 2. We systematically evaluate the performance of various baseline ReCDS systems, and the experimental results show that both ReCDS-PAR and ReCDS-PPR are challenging tasks. We also present relevant case studies to demonstrate the potential application and significance of our retrieval tasks in three typical clinical scenarios.

In summary, we introduce PMC-Patients, a first-of-its-kind dataset consisting of 167k patient summaries annotated with 3.1 M relevant patient-article pairs and 293k similar patient-patient pairs, serving as both a large-scale, high-quality, and diverse patient collection, and the largest-scale resources to benchmark ReCDS systems.

## Methods

In this section, we first describe the dataset collection process in detail. Then, based on PMC-Patients, we formally define two ReCDS benchmarks: Patient-to-Article Retrieval (ReCDS-PAR) and Patient-to-Patient Retrieval (ReCDS-PPR), and introduce the baseline ReCDS systems that we evaluate in this article.

### PMC-Patients dataset

**Fig. 3:**
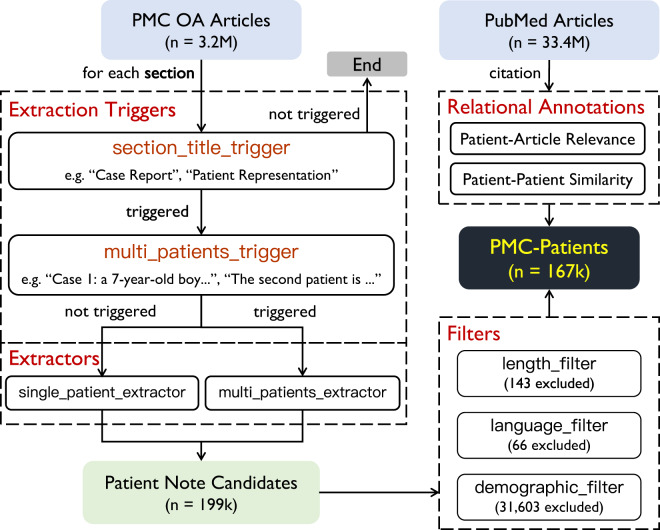
Collection pipeline of PMC-Patients. Patient summaries are identified by **extraction triggers**, extracted by **extractors**, and pass various **filters**. **Patient-level relations** are annotated using citation relationships in PubMed.

To collect the PMC-Patients dataset, we utilize the full-text literature resources in PubMed Central (PMC, https://www.ncbi.nlm.nih.gov/pmc/) and the citation relationships in PubMed (https://pubmed.ncbi.nlm.nih.gov/). We use the PMC and PubMed resources updated until Jan 7, 2022 via official FTP service (https://ftp.ncbi.nlm.nih.gov/pubmed/baseline/). We only use PMC articles with at least CC BY-NC-SA license (about 3.2 M) to build the redistributable PMC-Patients dataset. We do not exclusively use articles tagged as “case report” since we spotted numerous high-quality case reports in other types of articles in our pilot study. The collection pipeline can be summarized in three steps, as shown in Fig. 3:

- We identify potential patient summaries in each article section using **extraction triggers**.
- For sections identified above, we extract patient summary candidates using several **extractors**. Besides, we also extract the candidates’ demographics (ages and genders) using regular expressions.
- We apply various **filters** to rule out noises.

We then elaborate the technical details of the above steps.

#### Extraction triggers

Extraction triggers are a set of regular expressions to identify whether there are no, one, or multiple potential patient summaries in a given section, basically consisting of two successive triggers:

section_title_trigger: Searches in the section title for certain phrases that indicate the presence of patient summaries, such as “Case Report” and “Patient Representation”.

multi_patients_trigger: Searches for certain patterns in the first sentence of each paragraph and the titles of the subsections to identify whether multiple patients are presented, such as “The second patient” and “Case 1”.

Only articles with one or more potential patient summaries enter the following steps.

#### Extractors

Extractors perform at the paragraph level, i.e. an extracted patient summary always consists of one or several complete paragraphs and no split within a paragraph is performed. Depending on whether multi_patients_trigger is triggered, different extractors are used:

single_patient_extractor: Extracts all paragraphs in the section as one patient summary, if multi_patients_trigger is not triggered.

multi_patients_extractor: Extracts paragraphs between successive triggering parts (the last one is taken till the end of the section) as multiple patient summaries, if multi_patients_trigger is triggered.

#### Filters

We remove noisy candidates with three filters:

length_filter: Excludes candidates with less than 10 words.

language_filter: Excludes candidates with more than 3% non-English characters.

demographic_filter: Identifies the age and gender of a patient using regular expressions and excludes candidates missing either demographic characteristic.

In addition to the three filters described above, we incorporate an optional Humans_MeSH_filter. This filter is designed to eliminate candidates lacking the “Humans” MeSH term, primarily to exclude a minority of veterinary cases. However, this filter can only be applied to about one fourth of the candidates, as not all articles are tagged with MeSH terms. Considering the significance of both the dataset’s scale and quality, we opt to omit the application of the Humans_MeSH_filter in this paper, and introduce the resulting dataset with the filter applied, denoted as PMC-Patients-Humans, in Supplementary File 1. Further discussion on the issue of non-human cases will be presented in the “Dataset quality evaluation” section.

The regular expression rules and parameters in the modules above are generated empirically by manually reading and summarizing hundreds of case reports and then refined on a test set of 100 randomly selected articles.

#### Relational annotations

For each extracted patient summary in PMC-Patients, we use the citation graph of PubMed to automatically annotate (1) *relevant articles* in PubMed and (2) *similar patients* in PMC-Patients. Furthermore, we develop a 3-point grading system for both annotations, which may be more informative and reasonable.

##### Annotating relevant articles

We assume that if a PubMed article cites or is cited by a patient-containing article, the article is relevant to the patient. Besides, if the relevant article contains patient notes itself, we assume a higher relevance. Formally, we denote a patient as *p*, the article that contains *p* as *a*(*p*), and the collection of all patient-containing articles as ℕ. We define any PubMed article *a*′ moderately relevant to the patient *p*, denoted as $Rel(p,a{\prime} )=1$, if: $a{\prime} \mathop{\to }\limits^{{\rm{cites}}}a(p)$, or $a(p)\mathop{\to }\limits^{{\rm{cites}}}a{\prime}$, under the condition that *a*′ ∉ ℕ. We define *a*′ highly relevant to the patient *p*, denoted as $Rel(p,a{\prime} )=2$, if: $a{\prime} \mathop{\to }\limits^{{\rm{cites}}}a(p)$, or $a(p)\mathop{\to }\limits^{{\rm{cites}}}a{\prime}$, or $a(p)=a{\prime}$ (to ensure label completeness), under the condition that *a*′∈ℕ.

##### Annotating similar patients

We annotate similar patients based on relevant articles. For each patient in PMC-Patients, if its relevant articles contain other patients in the dataset, we will label them as similar patients, and patients extracted from the same article are given a higher similarity score. Formally, we define any two patients *p*_*x*_ and *p*_*y*_ in PMC-Patients moderately similar, denoted as $Sim({p}_{x},{p}_{y})=1$, if: $a({p}_{x})\mathop{\to }\limits^{{\rm{cites}}}a({p}_{y})$, or $a({p}_{y})\mathop{\to }\limits^{{\rm{cites}}}a({p}_{x})$, and highly similar, denoted as $Sim({p}_{x},{p}_{y})=2$ if $a({p}_{x})=a({p}_{y})$.

### PMC-Patients ReCDS benchmarks

Based on the patient summaries and relational annotations in PMC-Patients, we define two benchmarking tasks for ReCDS: Patient-to-Article Retrieval (ReCDS-PAR) and Patient-to-Patient Retrieval (ReCDS-PPR). Both are modeled as information retrieval tasks where the input is a patient summary *p* ∈ ℙ, where ℙ denotes the PMC-Patients dataset. For ReCDS-PAR, the goal is to retrieve PubMed articles relevant to the input patient using their titles and abstracts from the corpus 𝔸. Instead of using the entire collection of articles in PubMed, we narrow down the retrieval corpus to only include articles meeting the following criteria: 1) having machine-readable title and abstract; 2) in English; 3) tagged with “Humans” MeSH term. The restriction allows for more efficient dense retrieval, as encoding the whole PubMed collection would be time-consuming. Meanwhile, it reflects a practical search scenario where researchers often use similar filters to refine their searches. The resulting corpus 𝔸 consists of 11.7 M articles, a satisfying balance between efficiency and fidelity. For ReCDS-PPR, the objective is to retrieve patients similar to the input patient from PMC-Patients.

We split the train/dev/test sets on the article level. Specifically, we randomly select two subsets of articles (5k in each) from all patient-containing articles, and include the corresponding patients in the dev and test set as query patients. Patient summaries extracted from other articles are included as the training query patients and also used as the retrieval corpus ℙ for ReCDS-PPR. The benchmark statistics are shown in Table 1. It is worth noting that ReCDS-PPR dev and test sets do not contain highly similar patient annotations. This is because for each query patient in dev/test set, patients extracted from the same article, if any, will also be allocated to the dev/test split and thus not present in ReCDS-PPR corpus.

**Table 1:** Statistics of the ReCDS-PAR and ReCDS-PPR benchmarks.

Split	Source Articles	ReCDS-PAR			ReCDS-PPR		
		Query	Rel. 1/2	Avg. 1/2	Query	Sim. 1/2	Avg. 1/2
train	131k	154.5k	1.9 M/74.9k	12.3/0.5	94.6k	167.8k/89.5k	1.8/0.9
dev	5k	5.9k	70.6k/2.8k	12.0/0.5	2.9k	6.4k/0	2.2/0
test	5k	5.9k	74.1k/3.1k	12.5/0.5	2.8k	7.5k/0	2.7/0
**Corpus**		11.7 M candidate articles			155.2k candidate patients		

Rel. 1/2: numbers of relevant articles with score 1 and 2. Sim. 1/2: numbers of similar patients with score 1 and 2. Avg. 1/2: average numbers of annotations per query with score 1 and 2.

We evaluate retrieval models on both benchmarks using 3-point grades defined in the section above, with Mean Reciprocal Rank (MRR), Precision at 10 (P@10), normalized Discounted Cumulative Gain at 10 (nDCG@10), and Recall at 1k (R@1k).

### Baseline models

We implement three types of baseline retrieval models for both ReCDS-PAR and ReCDS-PPR: sparse retriever, dense retriever, and nearest neighbor retriever. Besides, in order to leverage both lexical and semantic match, which has been shown to further boost retrieval performance^21,22^, we use reciprocal rank fusion^23^ algorithm to combine sparse and dense retrievers.

#### Sparse retriever

We implement a BM25 retriever^24^ with Elasticsearch (https://www.elastic.co/elasticsearch). The parameters of the BM25 algorithm are set as default values in Elasticsearch (*b* = 0.75, *k*_1_ = 1.2). For ReCDS-PAR, we index the title and abstract of a PubMed article as separate fields and the weights given to the two fields when retrieving are empirically set as 3:1.

#### Dense retriever

Dense retrievers represent the patients and articles in a low dimensional space using BERT-based encoders and perform retrieval based on maximum inner-product search. Concretely, we denote the encoder as *f*, and **e**_*d*_ = *f*(*d*) refers to the low-dimensional embedding generated by the encoder for a given passage *d*. In our implementation, we take the embedding of the “[CLS]” token from the last layer as **e**_*d*_. Then for a query patient *q* and an article *a* in our retrieval corpus 𝔸, the relevance score between them is defined as the inner product of their embeddings: ${s}_{{\rm{dense}}}(q,a)={{\bf{e}}}_{q}\cdot {{\bf{e}}}_{a}$. The similarity score ${s}_{{\rm{dense}}}(q,p)$ between *q* and a patient *p* ∈ ℙ is defined similarly.

We first try direct transferring of bge-base-en-v1.5^25^ and MedCPT^26^, the state-of-the-art embedding models in general and biomedical domain, respectively. They are evaluated under a zero-shot setting, without further fine-tuning on PMC-Patients. Then we train our own dense retrievers by fine-tuning pre-trained encoders on the PMC-Patients dataset. To be specific, for a given query patient *q*_*i*_, a similar patient/relevant article ${p}_{i}^{+}$, and a set of dissimilar patients/irrelevant articles ${p}_{i,1}^{-},{p}_{i,2}^{-},\ldots ,{p}_{i,n}^{-}$ from the training data, we use the negative log-likelihood of the positive passage as the loss function:

1 $$L({q}_{i},{p}_{i}^{+},{p}_{i,1}^{-},{p}_{i,2}^{-},\ldots ,{p}_{i,n}^{-})=-log\frac{{e}^{{s}_{{\rm{dense}}}({q}_{i},{p}_{i}^{+})}}{{e}^{{s}_{{\rm{dense}}}({q}_{i},{p}_{i}^{+})}+\mathop{\sum }\limits_{j=1}^{n}{e}^{{s}_{{\rm{dense}}}({q}_{i},{p}_{i,j}^{-})}}$$

We train the dense retrievers with in-batch negatives^27^, where ${p}_{i,j}^{-}\in \{{p}_{k}^{+}\;| \;k\ne i\}$. Here we do not differentiate between the two levels of relevance/similarity scores since it is non-trivial to fully utilize such listwise information in dense retrievers^28^. We leave this for further work.

We train several different encoders, all of which are Transformer encoders^29^ initialized by domain-specific BERT^30^, including PubMedBERT^31^, BioLinkBERT^32^, and SPECTER^33^. For the ReCDS-PPR task, only one encoder is used, while for the ReCDS-PAR task, we train two independent encoders to encode patients and articles separately, due to their structural differences. In cases where the input text exceeds the maximum sequence length allowed by BERT models, we simply truncate it.

We implement all dense retrievers using PyTorch (https://pytorch.org/) and Hugging Face Transformers library (https://huggingface.co/docs/transformers/index). We train all dense retrievers for 50k steps on two NVIDIA GeForce RTX 3090 GPUs, with a batch size of 12 per device so that the GPUs are utilized to their full capacity. Our learning rate is set to 2e-5 with a 0.1 warmup ratio and a linear learning rate scheduler. We use the AdamW^34^ optimizer with weight decay of 0.05. Additionally, we apply gradient accumulation of 4 steps.

#### Nearest neighbor (NN) retriever

We assume that if two patients are similar, then their respective relevant article and similar patient sets should have a high overlap degree, based on which we implement the following NN retriever similar to^35^. For each patient in the training queries *p* ∈ ℙ, we define its relevant article set as ${\mathbb{R}}(p)=\left\{a| a\in {\mathbb{A}},Rel(p,a) > 0\right\}$. For each query patient *q*, we first retrieve top *K* similar training patients ${p}_{1},{p}_{2},\ldots ,{p}_{K}\in {\mathbb{P}}$ as its nearest neighbors using BM25. We also try using fine-tuned dense retrievers which give suboptimal performance. We take the union of their relevant articles as the candidate set:

2 $${{\mathbb{C}}}_{{\rm{PAR}}}(q)={\mathbb{R}}({p}_{1})\cup {\mathbb{R}}({p}_{2})\cup \cdots \cup {\mathbb{R}}({p}_{K})$$

Then the candidate articles ${c}_{i}\in {{\mathbb{C}}}_{{\rm{PAR}}}(q)$ are ranked by relevance scores ${s}_{{\rm{NN,PAR}}}(q,{c}_{i})$ defined as:

3 $${s}_{{\rm{NN,PAR}}}(q,{c}_{i})=\mathop{\sum }\limits_{k=1}^{K}{s}_{{\rm{BM25}}}(q,{p}_{k})I\left\{{c}_{i}\in {\mathbb{R}}({p}_{k})\right\}$$

For ReCDS-PPR, we define the similar patient set of each training patient $p\in {\mathbb{P}}$ as ${\mathbb{S}}(p)=\left\{p{\prime} | p{\prime} \in {\mathbb{P}},Sim(p,p{\prime} ) > 0\right\}$. The candidate set and similarity scores used for ranking are defined as:

4 $${{\mathbb{C}}}_{{\rm{PPR}}}(q)={\mathbb{S}}({p}_{1})\cup {\mathbb{S}}({p}_{2})\cup \cdots \cup {\mathbb{S}}({p}_{K})$$

5 $${}_{{}^{S}{\rm{NN,PPR}}}(q,{c}_{i})=\mathop{\sum }\limits_{k=1}^{K}{s}_{{\rm{BM25}}}(q,{p}_{k})I\{{c}_{i}\in {\mathbb{S}}({p}_{k})\}$$

In practice, we dynamically set *K* to include at least 10k candidates in each ${\mathbb{C}}(q)$ in order to ensure a moderate size of candidate set for each query.

#### Reciprocal rank fusion (RRF)

RRF is an algorithm to combine the results of several retrievers and has demonstrated the potential to yield enhanced retrieval performances^36^. Concretely, given a set of *D* documents to be ranked and a set of ranking results *R* from different retrievers, each a permutation on $1\ldots | D|$, the RRF score for certain document *d* is computed as:

6 $${s}_{{\rm{RRF}}}(d)=\sum _{r\in R}\frac{1}{k+r(d)}$$

where *r*(*d*) is the rank of document *d* in ranking results *r*, and *k* is a hyperparameter. In practice, we try different combinations of sparse retriever and the best performing dense retrievers (we do not use the NN retrievers due to their poor performances), and tune the hyperparameter *k* on our dev set. A combination of sparse retriever, PubMedBERT-based, and BioLinkBERT-based dense retrievers achieves optimal performances when *k* = 100 for ReCDS-PAR, and *k* = 5 for ReCDS-PPR.

## Data Records

The PMC-Patients dataset and ReCDS benchmark is publicly available on both figshare^37^ (https://figshare.com/collections/PMC-Patients/6723465) and huggingface (https://huggingface.co/zhengyun21). The detailed dataset formats are as follows.

### PMC-Patients dataset

We use the articles in PMC Open Access (OA) subset with at least CC BY-NC-SA license, amounting to 3,180,413, to ensure the open access to PMC-Patients. Among them, 198,846 patient note candidates are identified and extracted, and 167,034 patient summaries pass all three filters. Using the method described in the section above, we annotate 3,113,505 relevant patient-article pairs and 293,316 similar patient-patient pairs.

Patient summaries are presented as a *json* file, which is a list of dictionaries with the following keys:

- patient_id: string. A continuous id of patients, starting from 0.
- patient_uid: string. Unique ID for each patient, with format PMID-x, where PMID is the PubMed IDentifier of the source article of the patient and x denotes index of the patient in source article.
- PMID: string. PMID for the source article.
- file_path: string. File path of the *xml* file of the source article.
- title: string. Title of the source article.
- patient: string. The patient summary text.
- age: list of tuples. Each entry is in format (value, unit) where value is a float number and unit indicates the age unit (“year”, “month”, “week”, “day” and “hour”). For example, [[1.0, “year”], [2.0, “month”]] indicates that the patient is a one-year- and two-month-old infant.
- gender: “M” or “F”. Male or female.
- relevant_articles: dict. The key is PMID of the relevant articles and the corresponding value is its relevance score (2 or 1 as defined in the “Methods” section).
- similar_patients: dict. The key is patient_uid of the similar patients and the corresponding value is its similarity score (2 or 1 as defined in the “Methods” section).

### PMC-Patients ReCDS benchmark

The PMC-Patients ReCDS benchmark is presented as retrieval tasks and the data format is the same as BEIR benchmark^38^ (https://github.com/beir-cellar/beir). To be specific, there are queries, corpus, and qrels (annotations). Detailed statistics of the benchmark dataset is shown in Table 1.

ReCDS-PAR and ReCDS-PPR tasks share the same query patient set and dataset split. For each split (train, dev, and test), queries are stored a *jsonl* file that contains a list of dictionaries, each with two fields:

- _id: unique query identifier represented by patient_uid.
- text: query text represented by patient summary text.

Corpus is shared by different splits. For ReCDS-PAR, the corpus contains 11.7 M PubMed articles, and for ReCDS-PPR, the corpus contains 155.2k reference patients from PMC-Patients. The corpus is also presented by a *jsonl* file that contains a list of dictionaries with three fields:

- _id: unique document identifier represented by PMID of the PubMed article in ReCDS-PAR, and patient_uid of the candidate patient in ReCDS-PPR.
- title:: title of the article in ReCDS-PAR, and empty string in ReCDS-PPR.
- text: abstract of the article in ReCDS-PAR, and patient summary text in ReCDS-PPR.

Qrels are TREC-style retrieval annotation files in *tsv* format. A qrels file contains three tab-separated columns, i.e. the query identifier, corpus identifier, and score in this order. The scores (2 or 1) indicate the relevance level in ReCDS-PAR or similarity level in ReCDS-PPR.

## Technical Validation

In this section, we first analyze the characteristics of the PMC-Patients dataset, including basic statistics and patient diversity. We then show the dataset is of high quality in terms of the summary extraction and the relation annotation with human evaluation. We also present the performance of baseline methods on the ReCDS-PAR and ReCDS-PPR benchmarks, illustrating the challenges of our proposed benchmark. Finally, we carry out three case studies to show how clinicians can benefit from PMC-Patients in various ways.

### Dataset characteristics

#### Scale

Table 2 shows the basic statistics of patient summaries in PMC-Patients, in comparison to MIMIC, the largest publicly available clinical notes dataset, and TREC CDS, a widely-used dataset for ReCDS. For MIMIC, we report the statistics of discharge summaries of both MIMIC-3 and MIMIC-4. For TREC CDS, we combine the data released in three years’ CDS tracks (2014–2016) and use the “description” fields. PMC-Patients contains 167k patient summaries extracted from 141k PMC articles, making it the largest patient summary dataset in terms of the number of patients, and the second largest in terms of the number of notes. Besides, PMC-Patients has 3.1 M patient-article relevance annotations, which is over 27× the size of TREC CDS (113k in total). PMC-Patients also provides the first large-scale patient-similarity annotations, consisting of 293k similar patient pairs.

**Table 2:** Statistics of PMC-Patients, in comparison to MIMIC (d.s.: discharge summaries), and TREC CDS (2014–2016).

Dataset	Count		Average Length (words)	Relations	
	Patients	Notes		Relevant Articles	Similar Patients
PMC-Patients (ours)	**167k**	167k	410	**3.1 M**	**293k**
MIMIC-3 (d.s.)	41k	60k	1,282	—	—
MIMIC-4 (d.s.)	146k	**332k**	**1,480**	—	—
TREC CDS (all)	90	90	92	113k	—

#### Length

**Fig. 4:**
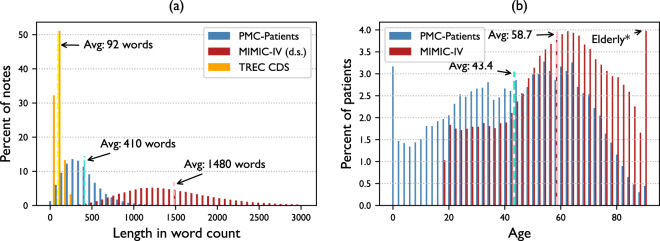
(**a**) Length distributions of PMC-Patients compared to MIMIC-4 discharge summaries and TREC CDS descriptions (x-axis truncated). (**b**) Patient age distributions of PMC-Patients compared to MIMIC-4. *Exact ages of patients older than 89 years old are obscured in MIMIC and thus taken as 90 in the figure.

In Fig. 4a, we display the length distributions of three datasets: PMC-Patients, TREC CDS descriptions, and MIMIC-4 discharge summaries. On average, PMC-Patients summaries are much longer than TREC descriptions (410 v.s. 92 words), but shorter than MIMIC discharge summaries (410 v.s. over 1k words). The differences in length among the three datasets can be attributed to their varying level of summarization. MIMIC provides comprehensive patient information as required for discharge summaries, while notes in PMC-Patients tend to focus mainly on the disease of interest, excluding irrelevant details. TREC, on the other hand, only summarizes core patient features for retrieval competition purposes.

#### Demographics

The age distributions of PMC-Patients and MIMIC-4 are presented in Fig. 4b. There are too few patients to observe the age distribution in TREC CDS, so we do not include it in the figure. On average, patients in PMC-Patients are younger than MIMIC-4 (43.4 v.s. 58.7 years old), and the quartiles indicate that PMC-Patients (Q1: 26; Q2: 45; Q3: 62) covers a wider range of patient ages while MIMIC-4 (Q1: 45; Q2: 60; Q3: 74) mainly focuses on those over 50. Besides, it can be observed from Fig. 4b that PMC-Patients covers pediatric patients while MIMIC-4 does not. The patient ages in PMC-Patients are more evenly distributed than MIMIC-4 (standard deviation: 22.7 v.s. 19.3 years; entropy: 6.39 v.s. 6.09 Shannon bits). The gender distribution in both datasets is balanced. PMC-Patients consists of 52.5% male and 47.5% female, while MIMIC-4 consists of 48.7% male and 51.3% female.

#### Medical conditions

**Fig. 5:**
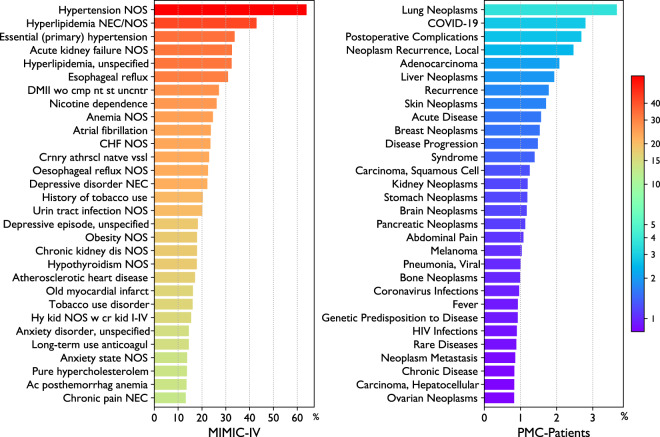
Relative frequency of top 30 ICD codes in MIMIC-IV (left) and MeSH Diseases terms in PMC-Patients (right). The colors are associated with relative frequency, and the color bar attached to the figure illustrates this.

We also analyze the medical conditions associated with the patients. For PMC-Patients, we use the MeSH Diseases terms of the articles, and for MIMIC, we use the ICD codes. The most frequent medical conditions are shown in Fig. 5. In PMC-Patients, the majority of frequent conditions are related to cancer, with the exception of COVID-19 as the second most frequent condition. In MIMIC-4, severe non-cancer diseases (e.g. hypertension) have the highest relative frequencies, and their absolute values are much higher than those of the most frequent conditions in PMC-Patients. For example, hypertension and lung neoplasms are the most frequent condition in MIMIC and PMC-Patients, respectively. Over 60% of MIMIC patients have hypertension, while less than 4% of patients in PMC-Patients have lung neoplasms. The significant difference between these two figures can be attributed to two factors. Firstly, PMC-Patients covers a broader range of diseases, including 4,031/4,933 (81.7%) MeSH Diseases terms, relatively more than MIMIC-4, which only comprises ICU patients and covers 8,955/14,666 (61.1%) ICD-9 codes and 16,464/95,109 (17.3%) ICD-10 codes. The nuanced distribution of MeSH Diseases terms occurrences in PMC-Patients can be found in Supplementary File 1. Secondly, the fact that PMC-Patients notes only focus on the disease of interest also constrain the MeSH terms to the specific disease, limiting the appearance of common comorbidities such as hypertension, the most frequent condition in MIMIC.

#### Relational annotations

**Fig. 6:**
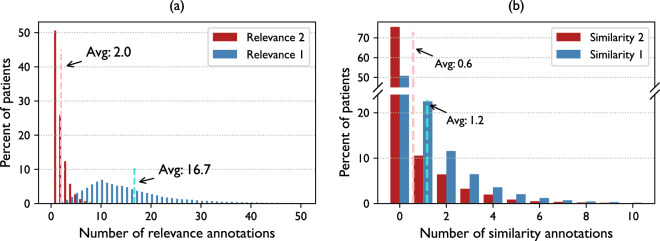
Distributions of (**a**) the numbers of relevance annotations and (**b**) the numbers of similarity annotations per patient. X-axis is truncated for both figures.

We further conduct statistical analysis to assess the degree of concentration of the annotated relations. Figure 6 displays the distributions of the number of relevance and similarity annotations per patient. As anticipated, the distributions exhibit significant skewness, especially for annotations with a score of 2. On average, each patient in PMC-Patients is associated with 2.0 highly relevant and 16.7 moderately relevant articles, along with 0.6 highly similar and 1.2 moderately similar patients.

### Dataset quality evaluation

#### Patient summary extraction

In this section, we evaluate the quality of the automatically extracted patient summaries and demographics in PMC-Patients. The evaluation is performed on a random sample of 500 articles from the benchmark test set. Two senior M.D. candidates are employed to label the patient note spans at the paragraph level and the patient demographics. Agreed annotations are directly considered as ground truth, while disagreed annotations are discussed until a final agreement is reached.

Table 3 shows the extraction quality of PMC-Patients and the two human experts against the ground truth. A total of 604 patients are extracted by human experts. The patient note spans extracted in PMC-Patients are of high quality with a larger than 90% strict F1 score. The extracted demographics are close to 100% correct. Besides, two annotators exhibit a high level of agreement, with most disagreements being minor differences regarding the boundary of a note span.

**Table 3:** Extraction quality of the PMC-Patients dataset and two experts against the ground truth.

Quality	Note Span	Age	Gender
PMC-Patients	91.24	99.77	100.0
Expert A	97.34	100.0	100.0
Expert B	97.28	99.91	99.49

Note span recognition is evaluated by F1 score. Age recognition is evaluated by min(annotated_age, true_age)/max(annotated_age, true_age). Gender recognition is evaluated by accuracy. All numbers are percentages.

#### Veterinary cases

To ensure a large dataset scale, we opt to forego the utilization of the Humans_MeSH_filter, thereby permitting the inclusion of certain veterinary cases in our dataset. In this section, we undertake an evaluation of the prevalence of such noises within two partition subsets of PMC-Patients. Firstly, among the 40,225 patient summaries possessing MeSH annotations, 39,764 are tagged with the “Humans” MeSH term, implying an approximate 1% incorporation of noise in this subset. The 39,764 patient summaries constitute PMC-Patients-Humans (the “purified” dataset with the Humans_MeSH_filter applied), whose detailed information can be found in Supplementary File 1. For those patient summaries lacking MeSH annotations, we manually inspect a sample of 100 patient notes and identify only one non-human case. Therefore, we posit that our dataset is reasonably compromised by a mere 1% of noise. This compromise is deemed acceptable, given the substantial augmentation in dataset scale, exceeding four times that of PMC-Patients-Humans.

#### Patient-level relation annotation

To evaluate the quality of patient-level relation annotations in PMC-Patients, we retrieve top 5 relevant articles and top 5 similar patients using BM25 for each patient extracted by the human experts in the previous section (604 patients from 500 articles), resulting in over 3k patient-article and 3k patient-patient pairs for human annotation. To annotate patient-article relevance, we follow the guidelines of the TREC CDS tracks^3,11,12^, where we annotate the type of clinical question that can be answered by an article about a patient, including diagnosis, test, and treatment. To annotate patient-patient similarity, we follow the recommendations from^14^, where we annotate whether two patients are similar in multiple dimensions: features, outcomes, and exposure. To assess the relational annotations in PMC-Patients against the multi-dimensional human annotations, we simply convert the latter into an integer score by counting the number of relevant or similar aspects. For example, if two patients are annotated as similar in terms of “features” and “outcomes”, we will give it a score of 2.

**Fig. 7:**
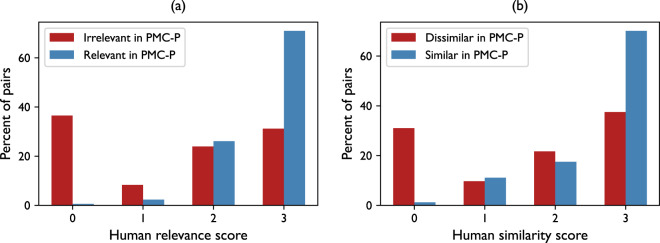
Distributions of (**a**) the human-annotated relevance scores and (**b**) the human-annotated similarity scores grouped by PMC-Patients automatic annotations.

Figure 7 shows the distributions of the human scores (x-axis) grouped by the relation annotations in PMC-Patients (binarized, Irrelevant v.s. Relevant and Dissimilar v.s. Similar). T-test shows that patient-article and patient-patient pairs with PMC-Patients annotations have significantly higher human scores than those without (*p* < 0.01 for both cases). Besides, almost all positive pairs are considered relevant/similar by a human expert, indicating PMC-Patients automatic relational annotations achieve quite high precision.

Still, it is important to recognize the presence of a minimal number of false positives in automatic relational annotations, such as background or methodological references. Potential solutions could entail devising more nuanced annotation weights based on the citation’s context and selectively excluding references to methodology articles. We leave these improvements for further work.

### ReCDS benchmark results

The performance of various baseline methods on the test set of two ReCDS tasks is presented in Table 4. Surprisingly, BM25 remains a strong baseline, yielding the best individual retriever performance in terms of MRR and nDCG@10 for the ReCDS-PPR task. Furthermore, it demonstrates competitive performance when compared to fine-tuned dense retrievers for the ReCDS-PAR task in terms of shallow metrics. This underscores the critical significance of exact word matching within case reports for the purpose of retrieving relevant articles or similar patients.

**Table 4:** PAR and PPR performances of baseline retrievers (in percentage).

Method	ReCDS-PAR				ReCDS-PPR			
	MRR	Prec	nDCG	Recall	MRR	Prec	nDCG	Recall
BM25	18.71	3.84	7.38	21.89	22.86	4.67	18.29	69.66
Dense retriever (zero-shot)								
bge-base-en-v1.5	15.88	4.27	6.44	30.43	16.20	3.78	13.02	68.85
MedCPT	13.06	2.67	4.95	19.94	13.68	3.18	11.01	60.17
Dense retrisever (fine-tuned)								
PubMedBERT	19.83	6.51	8.87	46.23	19.37	5.05	16.30	79.35
BioLinkBERT	19.06	6.11	8.26	45.79	21.20	5.59	18.06	80.49
SPECTER	17.92	5.49	7.66	42.46	15.08	3.79	12.27	73.01
NN retriever	16.45	4.98	6.43	30.93	6.30	2.40	4.83	59.49
RRF	**29.86**	**8.86**	**13.36**	**49.45**	**27.76**	**6.96**	**24.12**	**85.14**

Numbers in bold and underlined indicate the best and the second best results in each column, respectively. Precision (Prec) and nDCG are calculated at 10, and recall is calculated at 1,000.

Conversely, both bge-base-en-v1.5 and MedCPT, despite their record-setting performances in a multitude of embedding tasks, exhibit a notable lack of adaptability in the context of our ReCDS tasks. The shortcomings, particularly those of MedCPT, which is trained on PubMed search logs, highlight the distinctive and formidable nature of the ReCDS tasks, and further reveal the unique value of our dataset.

On the other hand, dense retrievers fine-tuned on PMC-Patients exhibit substantial improvements in performance, signifying the pivotal role of task-specific fine-tuning. While BM25 performs better on ReCDS-PPR in terms of shallow metrics, fine-tuned retrievers achieve the most elevated performance levels on ReCDS-PAR. Furthermore, they achieve significantly higher recall rates when compared to BM25, which is hindered by vocabulary mismatch. This affirms that semantic matching is an indispensable element in the retrieval of relevant articles and similar patients. Among the fine-tuned dense retrievers, PubMedBERT outperforms others on ReCDS-PAR, while BioLinkBERT achieves the best results on ReCDS-PPR. This superiority can be attributed to the distinctive pre-training corpus and tasks associated with these encoders: PubMedBERT and BioLinkBERT are both pre-trained on PubMed, with BioLinkBERT further incorporating citation graph data during its pre-training. SPECTER, despite also encompassing citation information, is pre-trained on general domain scientific literature, and thus performs less favorably on ReCDS tasks.

The NN retriever generally lags behind BM25 and dense retrievers on both tasks, suggesting that evaluating the relevance between patients and articles based on citation graph distance may not be a suitable approach for this task.

The RRF method, which combines the merits of both sparse and dense retrievers, delivers a substantial enhancement across all metrics for both tasks. Notably, the hybrid retriever elevates MRR and nDCG by nearly 50% over the top-performing individual retriever on ReCDS-PAR, and also achieves about 30% improvement in MRR and nDCG on ReCDS-PPR. Nevertheless, the metrics, despite the improvements, remain relatively modest, highlighting the challenge of the PMC-Patients ReCDS benchmark.

In summary, both ReCDS-PAR and ReCDS-PPR represent challenging endeavors, calling for further advancements in research.

### Case study

ReCDS provides valuable insights for healthcare providers in diagnosis, testing, and treatment of a queried patient, particularly in medically grey zones where high-level evidence is scarce, personalized management for multiple active comorbidities, and off-label use of novel therapeutics. We here present three case studies in the following section to demonstrate how PMC-Patients can benefit clinicians in various ways. Specifically, we focus on retrieval of similar patients since this is much less explored than relevant article retrieval. The three cases are selected and adapted from publications and TREC CDS 2016 patient collection, corresponding to three typical scenarios employed in TREC CDS: diagnosis (determining the patient’s diagnosis), test (determining what tests should the patient receive), and treatment (determining how should the patient be treated). For each case, we retrieve top 5 similar patients from our dataset using the BM25 retriever. Table 5 shows in brief the three cases with input summaries, examples of similar patients retrieved from PMC-Patients, and demonstrations of the clinical significance. The detailed inputs and outputs for performing case studies are shown in Supplementary File 2.

**Table 5:** Case studies on three patients under different scenarios.

Input summary	Retrieval output example	Description and significance
**Patient**: idiopathic thrombocytopenia, glomerulonephritis, and hearing impairment. Scenario: diagnosis	Case Report: Pathogenic MYH9 c.5797delC Mutation in a Patient With Apparent Thrombocytopenia and Nephropathy. (patient_uid: 8355614-1)	Identifying highly-likely combination of associated manifestation and underlying etiology for rare disease like field-experts
**Patient**: history of atrial fibrillation and deep vein thrombosis, signs of cholangitis. Scenario: test	Hemorrhagic cholecystitis causing hemobilia and common bile duct obstruction. (patient_uid: 6463387-1)	Highlighting related active issues for patients with multiple comorbidities thus overcoming cognitive blind-spot
**Patient**: melanoma, initially responsive to BRAF inhibitor but later progressed despite treated with PD-1 inhibitor. Scenario: treatment	Response to Ipilimumab/Nivolumab Rechallenge and BRAF Inhibitor/MEK Inhibitor Rechallenge in a Patient with Advanced Metastatic Melanoma Previously Treated with BRAF Targeted Therapy and Immunotherapy. (patient_uid: 7334770-1)	Out-of-textbook treatment for disease failing standard-of-care, thereby advancing implementation of off-label therapeutics

For each query patient, we present an example of the retrieved similar patients from PMC-Patients, with corresponding description and significance of assistance in query-patient management.

The first case involves a diagnostic dilemma of early-onset idiopathic thrombocytopenia, with co-occurred, seemingly unrelated conditions of renal disease, hearing loss, and suspicious family history. The top retrieved patient shows *MYH9* mutation^39^, which is the exact etiology of this case. *MYH9*-related thrombocytopenia is extremely rare (1:20,000–25,000)^40^ and is thus challenging to diagnose for non-experts. Other retrieval results also show other possible diagnoses including Alport syndrome^41^ and anti-basement membrane disease^42^. Its capability to recognize associated features from multiple manifestations and proposing insightful diagnoses is therefore greatly useful, especially in rare diseases.

The second case presents a female patient with a history of atrial fibrillation and deep venous thrombosis who shows acute hepatobiliary symptoms. ReCDS retrieves highly relevant cases, covering most common conditions including cholecystitis^43^, bile leak^44^, and Mirizzi syndrome^45^. Impressively, ReCDS is able to bring up potentially dangerous bleeding complications (hemobilia), via suspecting anticoagulation use from her cardiac and thrombotic comorbidities^46^ This requires further monitoring and testing, thus standing as important reminder in busy clinics where non-major medical problems can be easily ignored.

The third case asks an open question for treatment of metastatic melanoma failing standard care, pursuing answers in precision medicine similarly as the TREC PM 2020 track^47^. The retrieved cases include attempts of ipilimumab/nivolumab rechallenge, BRAFi and MEKi rechallenge^48^, and single agent PD-1 inhibitor^49^, each of which providing sound evidence with detailed clinical course background for an oncologist’s reference. Additionally, the approach itself favors effective treatment combinations (paradoxically thanks to positive report bias), and thus dynamically encourages evidence accumulation towards more promising directions, facilitating future clinical trial designs.

In conclusion, ReCDS can benefit clinicians in various ways, by recognizing rare diseases, overcoming testing blind spots, and advancing treatment evidence. With its potential to improve quality of medical care, ReCDS is especially valuable for clinicians in this era of precision medicine and personalized health.

### Supplementary information

Supplementary File 1

Supplementary File 2

## Data Availability

The code for collection PMC-Patients dataset and benchmark, as well as the code for reproducing the baseline models implemented in this paper is available at https://github.com/zhao-zy15/PMC-Patients. There is also a leaderboard of PMC-Patients benchmarks available at https://pmc-patients.github.io/. For those who are interested in improving ReCDS performances, please refer to https://github.com/pmc-patients/pmc-patients for evaluation code and submission guidelines.
