# Supplementary material for: A large-scale dataset of patient summaries for retrieval-based clinical decision support systems
Source: Sci Data. 2023 Dec 18;10:909. doi: 10.1038/s41597-023-02814-8 (PMC10728216; doi:10.1038/s41597-023-02814-8)
Supplement: Supplementary file 1 [file 41597_2023_2814_MOESM1_ESM.pdf]

Supplementary File 1

In this supplementary file, we introduce PMC-Patients-Humans, a subset of PMC-Patients, which undergoes an additional Humans_MeSH_filter. The filter is designed to exclude candidates lacking the "Humans" MeSH term, and ideally, such a filter can enhance the dataset's quality control by leveraging expert annotations via MeSH terms. However, since not all articles in PMC come with MeSH annotations, the Humans_MeSH_filter is not applicable to all patient notes. Out of the 167k patient notes identified, only 40,225 patient notes are extracted from articles with MeSH annotations, among which 39,764 are tagged with the "Humans" MeSH term. In essence, this filter eliminates 1% of noise (non-human cases) at the cost of reducing the dataset's scale by 75%. Given the significance of dataset scale alongside quality, we only introduce PMC-Patients-Humans in this supplementary file for the researchers whose studies necessitate an absolutely pure human case reports dataset.

Data Record

PMC-Patients-Humans comprises 39,764 patient notes extracted from 32,277 articles with the "Humans" MeSH annotation. We provide patient_uid of the patients in PMC-Patients-Humans in "MeSH_Humans_patient_uids.json" which can be found in "PMC-Patients Meta Data" on both figshare (https://figshare.com/articles/dataset/PMC-Patients_Meta_Data/24512725?backTo=/collections/PMC-Patients/6723465) and huggingface (https://huggingface.co/datasets/zhengyun21/PMC-Patients-MetaData). The json file is a list of string, each of which is a patient_uid and the whole dataset can be easily obtained by selecting these patients from "PMC-Patients.json".

Dataset Characteristics

Demographics: With the help of MeSH terms, we are able to analyze the demographic distribution of PMC-Patients-Humans via expert annotations since MeSH also comprises age group and gender tags. However, in order to obtain the age distribution annotated by MeSH age groups, we have to first address the issue that the MeSH terms are annotated at the article level and there are cases when several age groups are assigned to the same article with multiple patient notes. For example, in the article https://pubmed.ncbi.nlm.nih.gov/30373564/, the authors reported three patients and the article was annotated with both "Child" and "Adult", making it difficult to determine the exact age distribution. Therefore, in the following

discussion, we only consider a subset of PMC-Patients-Humans consisting of 28,517 patients extracted from articles with a unique MeSH age annotation.

Figure S1 displays the distribution of age groups annotated using MeSH terms in this subset of PMC-Patients-Humans, along with the age group distribution of PMC-Patients obtained by converting the ages automatically extracted via regex into the corresponding group (for instance, if the extracted age is 74 years old, we convert it into the group “Aged”). As shown in Figure S1, the distributions obtained via MeSH terms and regex extraction demonstrate a high level of agreement.

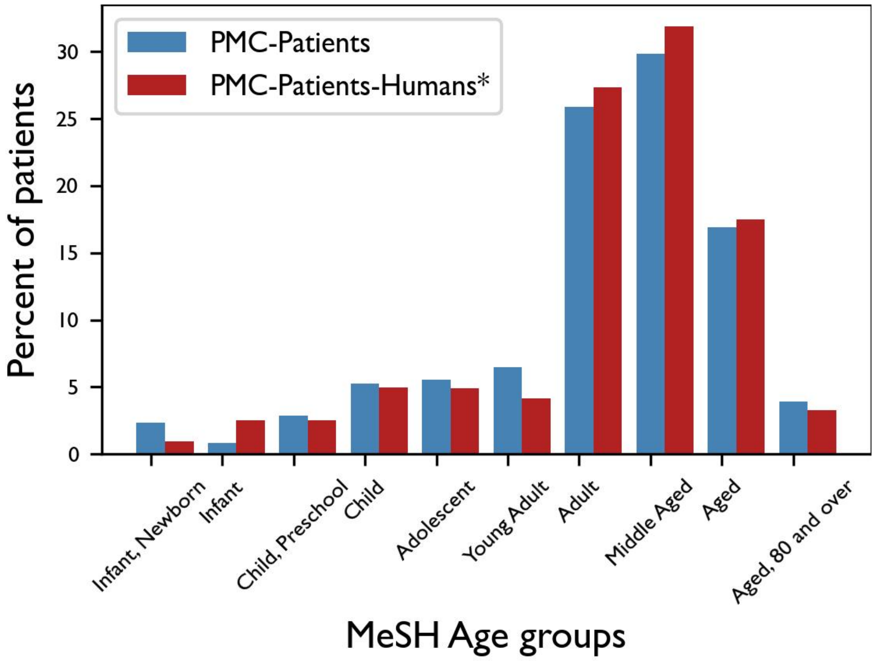

Figure S1. The distributions of age groups according to MeSH terms of PMC-Patients and PMC-Patients-Humans. The age groups of PMC-Patients are obtained by converting the exact ages extracted via regular expression into the corresponding group. * The subset of PMC-Patients-Humans consisting of 28,517 patients extracted from articles with a unique MeSH age annotation.

For gender distribution, we only consider the articles with a unique gender MeSH term, which amounts to 30,516, as well. PMC-Patients-Humans consists of 52.5% male, which is consistent with PMC-Patients.

To further investigate the demographic consistency of MeSH terms and regex extraction, we use the MeSH annotations as golden labels and calculate the accuracy of regex extraction in PMC-Patients-Humans. For example, if the extracted age is 74 years old, it is considered as correct if the article reporting the patient is

tagged with the MeSH term "Aged". Regex extraction achieves an accuracy of 96.6% and 99.2% in age and gender annotation, respectively. The results indicate a high level of agreement between our demographic extraction and the manual annotations via MeSH terms, which serve as additional warrant of the quality of our automatic annotations.

Medical conditions: MeSH thesaurus (https://meshb.nlm.nih.gov/treeView) is hierarchical-organized and thus allows for nuanced analysis at different hierarchy level. Figure S2 presents the distribution of first-level disease codes (C01-C26) in PMC-Patients and MeSH tree hierarchy. The distribution of MeSH terms occurred in our dataset roughly resembles the distribution of MeSH tree hierarchy, which indicates that PMC-Patients might be representative of a real patient note database in terms of the disease distribution. Table S1 shows the coverage rates of the MeSH diseases codes at different hierarchy level. It can be observed that PMC-Patients achieves a quite high-level coverage of MeSH diseases codes.

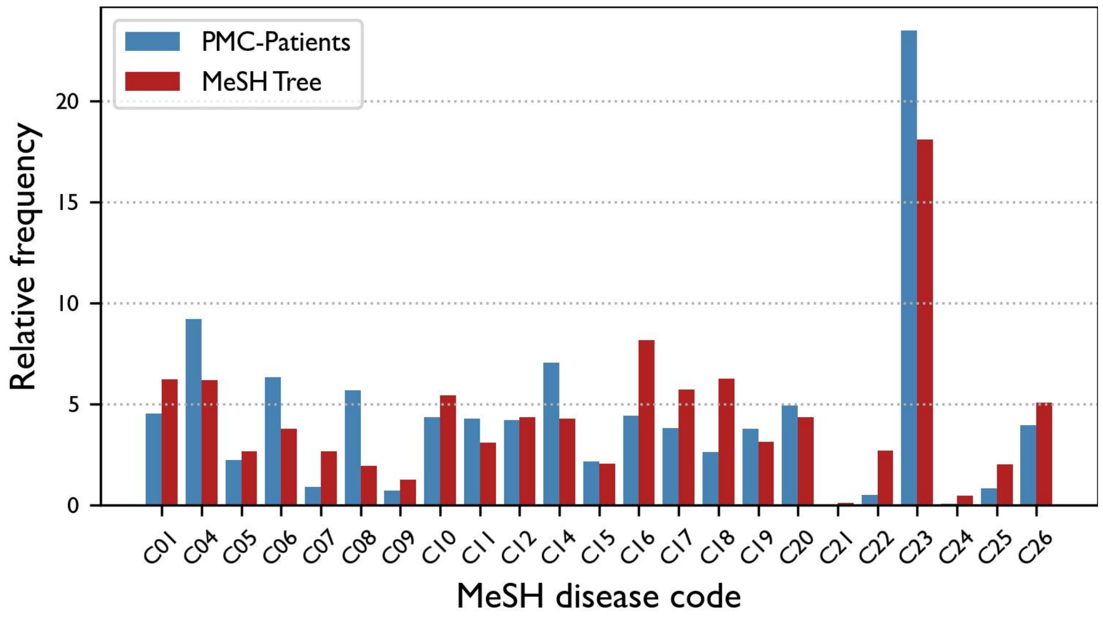

Figure S2. Distributions of MeSH Diseases codes (C01-C26) in PMC-Patients and MeSH tree hierarchy.

MeSH Tree Hierarchy*	#. of covered / total codes	Coverage(%)
3	23 / 23	100.0
6	219 / 238	92.0
9	889 / 1013	87.8
12	1358 / 1539	88.2
15	1106 / 1268	87.2
18	486 / 574	84.7

Table S1. Coverage rates of MeSH Diseases codes in PMC-Patients at different MeSH tree hierarchy. * MeSH tree hierarchy is represented by the number of the digits of each code. For example, "C01" is a 3-digit code, and "C21.223.500" is a 9-digit code. Codes with more than 18 digits are omitted due to their sparsity.
