# Supplementary material for: A large-scale dataset of patient summaries for retrieval-based clinical decision support systems
Source: Sci Data. 2023 Dec 18;10:909. doi: 10.1038/s41597-023-02814-8 (PMC10728216; doi:10.1038/s41597-023-02814-8)
Supplement: Supplementary file 2 [file 41597_2023_2814_MOESM2_ESM.pdf]

Supplementary File 2

In this supplementary file, we provide a comprehensive description of our case studies, including the source, full text of the input summary, and the top 5 similar patient results. We utilize the input format employed in TREC CDS descriptions, which involves a succinct and manual summary of patient conditions. This format allows us to emulate both TREC settings and real-world scenarios. The input summaries, except for case 2, are manually adapted and summarized by an M.D. candidate.

Case 1

Case 1 is adapted from https://www.ncbi.nlm.nih.gov/pmc/articles/PMC2428365/, which is not contained in PMC-Patients since the article does not have digital full-text resource.

Input summary: 'A 10-year-old girl presents with thrombocytopenia, hematuria and proteinuria indicating glomerulitis and decreased hearing. Multiple family members also had hearing impairments.'

Similar patients retrieved from PMC-Patients using BM25:

1. *Article PMID*: '34394193';
   Article title: 'Case Report: Pathogenic MYH9 c.5797delC Mutation in a Patient With Apparent Thrombocytopenia and Nephropathy';
   patient_uid: '8355614-1';
   Patient note: 'The patient was a 46-year-old Chinese woman. In November 2019, she was admitted to the department of hematology at our hospital due to thrombocytopenia, menorrhagia, and anemia. She was diagnosed with idiopathic thrombocytopenia purpura (ITP) and suspected inherited thrombocytopenia. Laboratory tests revealed a serum creatinine level of 158 \u03bcmol/L and trace urine protein without hematuria. As the main symptom was thrombocytopenia, and other manifestations were not recognized, she was treated for ITP. She received dexamethasone for 6 months, but her condition did not improve.\nIn May 2020, she received induced menopause treatment, and elevated serum creatinine and urine protein levels were found with no detailed information.\nIn June 2020, she was hospitalized again for further treatment at the local hospital; at this time, her serum creatinine level was 224 \u03bcmol/L, and urine protein was detected as \u201c2+\u201d with a quantitative test of urine protein indicating 2.4 g/day. Routine blood tests showed a significant decrease in the blood platelet count, which was 19 \u00d7 109/L (normal range, 100\u2013300 \u00d7 109/L), and her hemoglobin level was indicative of mild anemia. The results of pure tone audiometry indicated mild hearing loss and moderate hearing loss in the right and left ears, respectively. Considering the above results, MYH9-RD was suspected.\nShe was referred to the nephrology department for further diagnosis and therapy. Detailed examination revealed similar results as above: a platelet count of 27 \u00d7 109/L, urine protein level of \u201c3+,\u201d and serum creatinine level of 265 \u03bcmol/L [estimated

glomerular filtration rate (GFR), 18 ml/min). Another laboratory finding was elevated liver enzymes with an alanine aminotransferase (ALT) level of 78 U/L and aspartate aminotransferase (AST) level of 62 U/L. Ultrasound examination was suggestive of fatty liver and chronic kidney disease with cysts. Considering the characteristic manifestations of MYH9-RD described in the literature, a peripheral smear was performed, and typical inclusion bodies in the neutrophils were observed under a light microscope using Wright\u2013Giemsa staining (). Ophthalmic examination revealed binocular high myopia, while the refractive stroma of both eyes was transparent, indicating that the patient did not have typical MYH9-related ophthalmopathy, i.e., cataract. Renal puncture could not be performed because of the consistently low levels of blood platelets. Other medical history included hypertension for 6 years and diabetes mellitus for 1 week. Medications included levamlodipin beslate (2.5 mg) qd and repaglinide (0.5 mg) tid to control blood pressure and glucose. Her family history included thrombocytopenia (her mother).\nTo make a precise diagnosis, we performed a gene test for MYH9-RD related genes. Sequencing analysis revealed a heterozygous mutation c.5797delC (p. R1933Efs*15) in both the patient and her son in the MYH9 gene, but no mutations were found in the sister of the patient, who had normal manifestations (). The 23-year-old son of the patient, who appeared healthy, was found to have a decreased blood platelet count (62 \u00d7 109/L) and increased levels of liver enzymes (ALT 127 U/L, AST 54 U/L). Regarding renal function, the serum creatinine (67 \u03bcmol/L) and the GFR (GFR-cr 128 ml/min) were within normal range, but the urine protein fluctuated between \u201c2+\u201d and \u201c3+,\u201d but his hearing was not impaired. When tracing the history of the family, the mother of the proband, who was 71 years old, seemed to have MYH9-RD because she had thrombocytopenia (29 \u00d7 109/L) and severe deafness, which was later confirmed by whole-exome sequencing. After the clinical data of the pedigree members were collected, we noticed that the proband and her son shared similar disease spectrums related to MYH9-RD, with the exception of hearing loss, while her mother displayed a milder phenotype (). The family tree was drawn according to the clinical manifestations and sequencing results ().\nConsidering the physical and laboratory examinations mentioned above, our patient was diagnosed with chronic kidney disease stage IV, congenital thrombocytopenia (MYH9-related disease), hypertension, and diabetes.\nAfter admission, we initiated symptomatic treatment, including thrombopoietic drugs, antihypertensive drugs, and antidiabetic agents. The patient was educated to avoid drugs that affect platelet function to prevent bleeding. Intervention measures were taken for the son of the patient because he also had renal involvement. He was discharged with angiotensin receptor blockers (ARBs) to control proteinuria and body weight.\nAt her latest evaluation, 6 months after discharge, the serum creatinine level was stable at approximately 200 \u03bcmol/L with a quantitative urine protein level of 1.5 g/day and blood pressure of 126/78 mmHg. The serum creatinine level of the son of the patient was in the normal range, as before (64 \u03bcmol/L), while his proteinuria decreased with a quantitative urine protein level of 0.5 g/day.\nThe Global Variome database showed that the

c.5797delC mutation was pathogenic, but when we referred to the ClinVar database, we did not find a recorded item for this mutation. However, we found another substitution mutation at the same position, c.5795C>T, which was a known disease mutation in the ClinVar database. Web-based software Mutation Taster () predicted that this mutation was disease causing and capable of causing amino acid sequence changes, frameshift, and splice site changes (). In addition, arginine is an evolutionarily conserved amino acid in this protein among different organisms (), which indicates that changes in amino acids at this site may affect protein function.'

2. *Article PMID*: '26985371';
   Article title: 'Atypical anti-glomerular basement membrane disease';
   patient_uid: '4792615-1';
   Patient note: 'A 68-year-old Caucasian male underwent a renal allograft biopsy to evaluate gross hematuria and rising creatinine from 0.7 to 1.2 mg/dL, 3 months following transplantation (Table).\nHis past medical history included asymptomatic proteinuria at the age of 18 years discovered during a pre-employment assessment, not further evaluated. At the age of 61 years, he underwent right nephrectomy for renal cell carcinoma (clear cell type, 3.1 cm size, stage pT1a, NX). This was followed by recurrent urinary tract infections requiring prolonged antibiotic treatment, new onset hypertension and hematuria attributed at that time to a renal stone. His renal function progressively deteriorated over the following year, and he began hemodialysis. He had no family history of renal disease, hearing defects, hematuria or vision abnormalities.\nHe received a four antigen HLA mismatched kidney transplant from a standard criteria deceased donor after 52 months on dialysis. Graft function was excellent with creatinine of 0.7 mg/dL (basiliximab induction; standard tacrolimus, prednisone and mycophenolate mofetil immunosuppression). Persistent microscopic hematuria was detected on Postoperative Day (POD) 15, and, gross hematuria developed 1 week after ureteral stent removal. His urine contained dysmorphic red blood cells (RBCs) without casts; proteinuria (UPr:Cr) of 1.2 (up from 0.4), and serum creatinine rose to 1.2 mg/dL. Serologic studies including anti-GBM antibody were negative (Table). Imaging was unrevealing, and cystoscopy noted allograft origin of the hematuria. The recipient of contralateral kidney from the same donor had no hematuria. An allograft biopsy was performed at POD 83.\nHistopathologic findings are detailed in Table and illustrated in Figure . Unexpectedly, serial allograft biopsies studied by immunofluorescence showed ribbon-like linear staining of GBMs for IgG-lambda; further characterized as IgG3-lambda monotypic. Despite the linear staining and the presence of RBC casts, cellular crescents were not seen; only mild glomerulitis and focal mild endocapillary/mesangial hypercellularity were noted (Figure). There were no deposits seen by electron microscopy. Progressing interstitial fibrosis and tubular atrophy (IFTA) have been observed on multiple biopsies examined over a period of 12 months since transplantation, without evidence of rejection. The 1-year biopsy demonstrated a greater proportion of glomeruli involved by mild mesangial and endocapillary hypercellularity, along with two fibrocellular crescents.\nIn addition,

slides from the native nephrectomy specimen from 7 years earlier were obtained from the referring center for retrospective review (Figure). The findings included numerous RBC casts and mild segmental proliferation and glomerulitis, without crescents. There were no electron-dense or fibrillary deposits and no basement membrane changes of Alport's syndrome on retrospective electron microscopic analysis of the native nephrectomy.\nThe findings were interpreted as a form of monoclonal anti-GBM disease in the allograft. Furthermore, findings in the native nephrectomy specimen were concerning for the same process. Additional anti-GBM studies were pursued; serum from POD 100 was reported as positive by indirect immunofluorescence antibody testing [immunofluorescence assay (IFA)], but was negative by qualitative multi-analyte fluorescence detection. Sera from later time points, however, were repeatedly negative for anti-GBM by ELISA or IFA tests. Given the apparent monotypia of the deposits, further hematologic studies included serum and urine protein electrophoresis, evaluation of peripheral blood for free light chains and flow cytometric analysis of lymphocytes, all of which were negative for a clonal lymphoproliferative process. The patient was treated with plasmapheresis, intravenous immunoglobulin, steroids and a single dose of rituximab. Thereafter, he resumed his baseline triple immunosuppressive therapy, and despite increasing IFTA, maintains graft function (creatinine 1.0 mg/dL) with 2+ proteinuria and >50 RBCs on spot urinalysis, 56 months post-transplant.'

3. *Article PMID*: '32543079';
   Article title: 'Pathogenic evaluation of synonymous COL4A5 variants in X-linked Alport syndrome using a minigene assay';
   patient_uid: '7434753-1';
   Patient note: 'Patient 1, male, was 19 years old at the time of genetic analysis. Microhematuria and proteinuria had been noted for the patient from 3 years of age. Histological evaluation of a kidney biopsy at 6 years of age revealed lamellation of the GBM and negative expression of \u03b13/\u03b14/\u03b15 (IV). The patient's disease developed into end-stage renal disease (ESRD) at the age of 18 years. The patient's older brother had also been diagnosed with hematuria and proteinuria at the age of 2 and had an estimated glomerular filtration rate (eGFR) of 32.4 mL/min/1.73 m2 at the age of 23. Their mother had hypertension, microhematuria, and proteinuria (0.2 g/g Cr) and her eGFR was 60.7 mL/min/1.73 m2 at 49 years of age. None of the family members had ocular abnormalities or hearing loss. The clinical course and gene test results of this family have been previously reported by our group (Fu et al.,).'

4. *Article PMID*: '33363791';
   Article title: 'A case of hypoparathyroidism, deafness, and renal dysplasia (HDR) syndrome with a novel frameshift variant in GATA3, p.W10Cfs40, lacks kidney malformation';
   patient_uid: '7752573-1';

Patient note: 'A 24-year-old man was referred to our department for investigation of hypocalcemia. He was born from nonconsanguineous Japanese parents as a low birth weight immature baby of 1114 g with ptosis and joint contracture, although no neonatal asphyxia, cerebral dysgenesis, or multiple abnormality were described. His moderate bilateral sensorineural hearing loss was pointed out at the age of 4, resulting in requirement for a hearing aid. He often experienced facial spasm, tetany, as well as leg cramp, leading to falling down, at around the age 10, although those symptoms had been improved without intellectual disability during puberty. Since his tetany worsened again, he saw a primary physician, and pointed out hypocalcemia, (adjusted serum calcium, 6.1 mg/dL; reference interval 8.8-10.1) and hyperphosphatemia (5.4 mg/dL; 2.7-4.6). Oral administration of vitamin D and calcium lactate hydrate was started, resulting in serum calcium normalized and the symptoms improved. He had no family history of parathyroid diseases, tetany, deafness, kidney dysplasia, or other congenital anomalies.\nLaboratory tests (Table) demonstrated marked reduction of intact PTH to 15 pg/mL (reference interval: 18.5-88.0), but mild hypocalcemia with a serum calcium level of 8.3 mg/dL, and phosphorus within normal range at 4.9 mg/dL, due to the administrations of vitamin D and oral calcium supplement. Basal levels of other hormones were within the normal range. No evidence of renal dysfunction, proteinuria, hematuria, or urinary tract infection was obtained. Electrocardiography was negative for cardiac hypertrophy or ischemia. Neck ultrasound indicated no apparent nodule or abnormality in and around the thyroid glands. A brain CT showed multiple calcifications of basal nuclei (Figure), while abdominal CT demonstrated no morphological abnormality of kidneys (Figure).\nDifferential diagnosis of early-onset hypoparathyroidism includes several congenital diseases, DiGeorge syndrome, Kenny-Caffey syndrome, and a genetic abnormality in PTH or CaSR, although deafness is almost always observed in HDR syndrome and more rarely in DiGeorge syndrome. To distinguish between HDR syndrome and DiGeorge syndrome, we evaluated classification of leukocytes by flow cytometry (Table), resulting in a decreased ratio of Th2/CD4 + T cells to 0.3% (0.32-3.24), as well as an elevated ratio of Th1/Th2 to 76.2 (6.34-29.67). These indicated a reduced differentiation of naive CD4 + cells into Th2 lymphocytes, consistent with HDR syndrome. FISH analysis did not reveal any microdeletion in the 22q11.2 region, indicating less possibility of DiGeorge syndrome. Those results encouraged us to perform a single gene analysis of GATA3 with peripheral blood cells, even without renal anomaly, one of the three features of HDR syndrome. Informed consent for genetic studies was obtained from the patient and his family members, under the approval by the institutional ethical committee. The Sanger sequencing analysis demonstrated a heterozygous variant, c.30-49del-insCACCGAGCTGCA (Figure , upper panel), leading to a frameshift variant, p.Trp10Cysfs40. Pedigree analysis indicated no symptoms of HDR syndrome, as well as no pathogenic variant in GATA3, in both parents (Figure , middle and lower panels), indicating the insertion-deletion variant detected in the proband was de novo.\nTo perform genetic testing in his parents, several genetic counseling sessions were required for their acceptance. Disclosing the results,

however, much improved their mood and reduced their anxiety, without apparently affecting their relationship.'

5. *Article PMID*: '33994692';
   Article title: 'A Case of Hearing Impairment with Renal Dysfunction';
   patient_uid: '8101677-1';
   Patient note: 'A 46-year-old woman presented to hospital with complaints of nausea, vomiting, and generalized weakness since 2\u20133 weeks associated with swelling over body. On evaluation, she was found to have severe renal dysfunction and initiated on hemodialysis. Her urine examination revealed 1+ protein and 3-4 RBCs with urine PCR of 2.48. She was continued on maintenance hemodialysis elsewhere after placement of left-arm AV fistula.\nShe came to our hospital for renal transplantation with younger brother being prospective donor. On evaluation, both of them had history of hearing impairment since childhood. Detailed family history revealed history of hearing impairment in her father and another younger brother . Further investigations revealed sensorineural hearing loss and dot and fleck retinopathy in both of them. However, brother did not have proteinuria and/or renal dysfunction. Neither of them had any history of hematuria. In view of hereditary hearing impairment, retinopathy, and renal failure, possibility of Alport's syndrome was considered. Kidney biopsy was done which revealed nodular glomerulosclerosis with congophilic deposits in mesangium and arterioles which displayed apple-green birefringence on polarized light. IF was negative and kappa/Lambda did not show any restriction. Considering possibility of hereditary amyloidosis, genetic analysis was done which revealed mutation in NLRP3 gene in both patient and her brother. The variant identified was c.1049 C>T p.Thr350Met. This variant was found to be likely pathogenic for MWS, as per ACMG 2015 guidelines.[] This variant has also been reported by Kuemmerle-Deschner et al.[] in the cases of MWS. On retrospective enquiry, she also gave the history of episodes of cold-induced skin rash, arthralgia, and low-grade fevers. Hence, diagnosis of MWS was established. We offered genetic testing for other family members, but they have not consented for it.'

Case 2

Case 2 is directly taken from TREC 2016 CDS test patient collection, without modifying.

Input summary: 'A 94 year old female with hx recent PE/DVT, atrial fibrillation, CAD presents with fever and abdominal pain. An abdominal CT demonstrates a distended gallbladder with gallstones and biliary obstruction with several CBD stones.'

Similar patients retrieved from PMC-Patients using BM25:

1. *Article PMID*: '22375186';

Article title: 'The Successful Treatment of Chronic Cholecystitis with SpyGlass Cholangioscopy-Assisted Gallbladder Drainage and Irrigation through Self-Expandable Metal Stents';

patient_uid: '3286734-1';

Patient note: 'A 34-year-old female with a history of advanced pulmonary sarcoidosis and right-sided heart failure presented with chronic, postprandial right upper quadrant pain and weight loss. An abdominal ultrasound revealed cholelithiasis and choledocholithiasis with dilatation of the common bile duct (CBD). Endoscopic retrograde cholangiopancreatography (ERCP) revealed a distal CBD stricture with proximal dilatation of the biliary tree and several stones. The stricture was dilated with a 4 cm\u00d730 Fr biliary balloon dilatation catheter (Hurricane\u2122 RX; Boston Scientific, Cork, Ireland). Sphincterotomy was then performed and two CBD stones were removed with a 12/15 mm biliary retrieval balloon (Extractor\u2122 RX; Boston Scientific, Cork, Ireland). Repeat cholangiogram showed clearance of the CBD and hepatic ducts, however, numerous stones were seen in the gallbladder. Despite the patient's young age and persistent symptoms, cholecystectomy was deemed too high risk and was not performed. Endoscopic biliary drainage was deemed to be the most appropriate therapeutic option.\nRepeat ERCP utilizing the SpyGlass cholangioscopy system (SpyGlass Direct Visualization System; Microvasive Endoscopy, Boston Scientific Co., Natick, MA, USA), enabled direct visualization of the cystic duct and gallbladder, and a 0.035 in\u00d7260 cm guide wire (Hydra Jagwire\u2122; Boston Scientific, Miami, FL, USA) was placed into the gallbladder. On fluroscopy the cystic duct was approximately 4 mm in diameter and the 10 Fr Spy-Glass cholangioscopy system was easily advanced into the cystic duct, without need for dilatation. Given these measurements we estimated that the cystic duct would safely accommodate the 7 Fr delivery system of the biliary self expanding metal stents (SEMS). In order to traverse the entire length of the cystic duct, two overlapping 10\u00d780 mm fully covered self-expanding metal biliary stents (Wall-Flex\u2122 Biliary; Boston Scientific, Galway, Ireland) were placed over the guide wire into the gallbladder. Subsequently, a third 10\u00d780 mm fully covered SEMS (Wall-Flex\u2122 Biliary, Boston Scientific, Galway, Ireland) was placed into the CBD (). The distal CBD stricture appreciated on the first ERCP had resolved post biliary balloon dilatation and the three biliary stents were easily advanced into place. Numerous small stones were noted to drain from the gallbladder after stent placement. The gallbladder was then thoroughly irrigated by advancing the SpyScope with an attached irrigation system (EndoGater\u2122; Byrne Medical Inc., Conroe, TX, USA) resulting in clearance of additional stones (). Repeat cholangiogram revealed a contracted gallbladder with no filling defects in the CBD or the gallbladder. The patient was a poor surgical candidate, and in lieu of cholecystectomy, we chose fully covered metal stents to allow for the irrigation and removal of all the gallstones, as well as a longer period of drainage. Plastic biliary stents would allow for drainage, but not stone removal. The fully covered SEM biliary stents were chosen for the CBD and gallbladder in order to allow for the possibility of future removal. The stents were kept in place post irrigation to ensure continued

drainage.\nThe patient did well post procedure and was discharged home on a regular diet several days later. An abdominal ultrasound at one month follow-up revealed a normal appearing gallbladder without evidence of stones or sludge. At 5-month follow-up the patient had no abdominal complaints and had normal liver function tests. At 1-year follow-up, the patient was pain free but the plan for stent removal was halted by her ongoing pulmonary issues.'

2. *Article PMID*: '31001409';

Article title: 'Hemorrhagic cholecystitis causing hemobilia and common bile duct obstruction';

patient_uid: '6463387-1';

Patient note: 'A 78-year-old male with past medical history of atrial fibrillation (on Warfarin), Type 2 diabetes mellitus, hypertension, and coronary artery disease presented to the Emergency Department with a chief complaint of epigastric abdominal pain radiating to the central abdomen which was worsened with food intake. Associated symptoms included nausea, emesis, fever, and chills. On physical exam, he was noted to have epigastric tenderness, absent Murphy\u2019s sign, scleral icterus and an irregularly irregular heart rhythm. He did not demonstrate symptoms of gastrointestinal bleeding at the time of presentation.\nLaboratory results were as follows: white blood cell count 10.5, hemoglobin 13.9, platelet count 164, Total bilirubin 3.8, AST 133, ALT 200, alkaline phosphatase 339, lipase 33, protime 36.3, INR 4.2. Abdominal ultrasound was obtained to evaluate for potential gallbladder/biliary pathology given his presentation. The ultrasound demonstrated gallstones with findings concerning for chronic cholecystitis with a common bile duct measurement of 8 mm. To further evaluate the cause for the patient\u2019s abdominal pain, a computed tomography (CT) scan was performed of the abdomen and pelvis. CT findings demonstrated an abnormal gallbladder with stones and dense intraluminal debris measuring 53 Hounsfield units. Similar debris was seen in the common bile duct with a measurement of 17 Hounsfield units. Active contrast extravasation into gallbladder lumen was not seen on the CT, but Hounsfield units were consistent with intraluminal blood. The common bile duct was measured at 8 mm in diameter without intrahepatic ductal dilatation (Fig.).\nThe patient was admitted for management of obstructive jaundice with possible cholecystitis. On hospital day 1 his bilirubin increased to 5.1, he was given Vitamin K and fresh frozen plasma to correct his coagulopathy and he was taken for endoscopic retrograde cholangiopancreatography (ERCP). During ERCP, the common bile duct (CBD) was cannulated and swept revealing a moderate amount of maroon clot (Fig.). No other debris or stones were noted to be within the CBD on final fluoroscopic image (Fig.).\nOn hospital day 2, laboratory values improved overall with bilirubin of 2.1, AST 57, ALT 130 and Alkaline phosphatase of 254. The patient was then taken to the OR for laparoscopic cholecystectomy. Intraoperative findings included: dense omental adhesions, thickened gallbladder wall, extensive pericholecystic edema, cholelithiasis, and a large clot within the gallbladder lumen. Postoperatively, patient\u2019s recovery was uneventful with down trending direct bilirubin,

resolution of pain, and successful restarting of his Warfarin. He made a full recovery by his post-operative follow-up visit.'

3. *Article PMID*: '27445508';

Article title: 'Mirizzi syndrome with an unusual aberrant hepatic duct fistula: a case report';

patient_uid: '4938132-1';

Patient note: 'The patient, a 76-year-old Asian female, was detected with liver gallstones (lithiasis) by ultrasonic diagnosis during a health examination and was admitted to our department. At that time, she did not show fatigue, jaundice, intermittent high fever, and right upper quadrant abdominal pain. There was no significant medical background and family history. Moreover, according to the abdominal findings via physical examination, no icteric scleras and right upper quadrant abdominal tenderness in deep palpation were found, nor were the rebound tenderness or abdominal muscle rigidity and palpable masses found. The following were the testing indexes:\nBlood tests showed ALT: 313 U/L (normal: 0\u201340 U/L), AST: 443 U/L (normal: 0\u201340 U/L), ALP: 67 U/L (normal: <106 U/L), and \u03b3-GGT: 93 U/L (normal: <50 U/L) with normal bilirubin levels: 20 \u03bcmol/L (normal values for total bilirubin: 0\u201325 \u03bcmol/L). Serologic tests for hepatitis B and C were all negative. Tumor markers including \u03b1-fetoprotein, carcinoembryonic antigen, and carbohydrate antigen 19-9 were within normal limits. US examination showed cholelithiasis with a distended gallbladder, multiple calculi within its lumen, and moderate intrahepatic biliary dilatation. Abdominal enhanced multidetector computed tomography scan revealed an atrophic right posterior liver and a dilated hepatic duct and right posterior sectional duct with multiple stones inside. MRCP indicated the presence of a sinus tract between the gallbladder fossa and right posterior hepatic duct. Cholecystobiliary and aberrant right posterior hepatic duct fistula were also observed (). Computed tomographic (CT) scans showed multiple dilated bile ducts in the right posterior segment with stones ().\nAlthough the patient was suffering from severe lithiasis, she did not show any symptoms. On the basis of the aforementioned data, the initial diagnosis before operation was MS complicated with intrahepatic lithiasis associated with aberrant right posterior hepatic duct fistula. Surgery remained the exclusive choice of curative therapy, and the definitive diagnosis was confirmed intraoperatively. An exploratory laparotomy through a right subcostal incision was performed. During cholecystectomy, two severe anatomic alterations were observed. The first was an impact stone found in the cystic duct, which firmly adhered to the aberrant right posterior hepatic duct and the cholecystobiliary fistula, and involved less than one-third of the circumference of the duct. The other was a wide range of tissue adhesion, which was associated with elongated cystic duct, parallel to the CBD. Right posterior lobe was resected. Multiple cystic dilations of the intrahepatic biliary tree and black pigment stones adherent to the bile duct wall were observed in the resected specimen (). The CBD was lavaged, and the defect of the CBD was sutured in a one-layer manner. Finally, a T-tube was inserted. The postoperative course was

uneventful and the patient was discharged a week after the operation. Routine follow-up was continued in the hepatobiliary clinic.'

4. *Article PMID*: '32542169';
   Article title: 'Biloma: A Rare Manifestation of Spontaneous Bile Leak';
   patient_uid: '7292700-1';
   Patient note: 'A 91-year-old female presented to the emergency room with acute onset of epigastric and RUQ pain for one day. The pain was described as sharp, radiating to her back, and associated with two to three episodes of non-biliary, non-bloody vomiting. She denied fever, bowel movement irregularities, weight loss, abdominal trauma, or prior abdominal surgeries. Clinical examination showed RUQ tenderness without rebound tenderness or Murphy\u2019s sign. Laboratory workup was significant for elevated levels of lipase (5,700 U/L), lactic acid (2.2 mmol/L), and creatinine (1.71 mg/dL). A complete blood count, liver function tests (total bilirubin 0.2 mg/dL, alanine aminotransferase 9 U/L, aspartate aminotransferase 12 U/L, alkaline phosphate level 94 U/L), and alpha-fetoprotein (AFP) tumor (1.3 IU/mL) were unremarkable. CT scan of the abdomen and pelvis revealed a distended gallbladder with wall thickening, dilated common bile duct (CBD), but without evidence of pancreatitis or gallstones (Figure).\nAbdominal US showed trace intramural and pericholecystic fluid with no abnormality of the CBD. HIDA scan findings were consistent with extrahepatic biliary leakage into the peritoneum (Figure).\nMRCP revealed moderate pericholecystic and perihepatic fluid collection (Figure).\nA cholangiogram demonstrated a perihepatic biloma, which was drained under the guidance of fluoroscopic imaging (Figure).\nOn endoscopic retrograde cholangiopancreatography (ERCP), there was no clear evidence of contrast extravasation; however, a blush of contrast at the junction of the cystic duct and the common hepatic duct was seen, which correlated with the location of biloma noted on the HIDA scan (Figure).\nSince there was clear evidence of bile in the percutaneous drain with no clinical or endoscopic evidence of the bile leak, a 10-French plastic stent was placed in the CBD across the junction of the bile duct with the cystic duct where a blush of contrast was seen on ERCP (Figure).\nThereafter, the patient\u2019s symptoms completely resolved, and she was discharged home. Upon follow-up five weeks after the intervention, a repeat ERCP showed that the bile leak had resolved, and the plastic stent was removed.'

5. *Article PMID*: '33613164';
   Article title: 'Biliary Peritonitis Caused by Spontaneous Bile Duct Rupture in the Left Triangular Ligament of the Liver after Endoscopic Sphincterotomy for Choledocholithiasis';
   patient_uid: '7879265-1';
   Patient note: 'A 91-year-old man presented with abdominal pain in the right upper quadrant. His medical history included chronic pancreatitis, chronic atrial fibrillation, and angina pectoris. He took apixaban (a factor Xa inhibitor, which is a direct oral anti-coagulant [DOAC]) for atrial fibrillation. His laboratory data showed inflammatory

reaction (white blood cells [WBCs], 10,200/\u03bcL; C-reactive protein [CRP], 3.34 mg/dL) and an increase in hepatobiliary enzyme and serum amylase levels. On his abdominal computed tomography (CT) scan, gallbladder stones and pancreatic stones, which likely resulted from chronic pancreatitis, and an increase in fat density around the pancreas were observed. He was diagnosed with acute exacerbation of chronic pancreatitis and immediately admitted to our institution where he was treated with fasting and a drip infusion. His symptoms disappeared and food intake resumed on day 4 after admission. The patient's laboratory test results showed a sustained increase in hepatobiliary enzymes and amylase (Fig.). On day 7, magnetic resonance cholangiopancreatography showed possible tiny biliary stones in the distal bile duct. Antibiotics (cefmetazole) were started. On day 11, ERCP revealed CBD stones, and an ERBD plastic stent was inserted (Fig.).\nAlthough EST was performed for the common bile duct, the patient's laboratory test results remained in the abnormal range (Fig.). On day 16, the inflammatory reaction was somewhat decreased (WBCs, 12,600/\u03bcL; CRP, 1.54 mg/dL). On day 17 (1 week after the initial ERCP), EST and removal of bile stones were performed and the ERBD was removed (Fig.). On day 18, food intake and DOAC resumed. On day 19, he complained of abdominal pain. An abdominal CT scan was performed to further investigate his symptoms and the elevated hepatobiliary enzymes (Fig.). The CT results showed retention of contrast medium in the gall bladder and CBD as well as fluid collection (approximately 5 cm in diameter) in the sub-hepatic area of the lateral segment. On day 21, his abdominal pain recurred. The patient's laboratory data showed that inflammatory markers were elevated (WBCs, 22,400/\u03bcL; CRP, 29.38 mg/dL). His abdominal CT scan showed fluid collection that was approximately 10 cm in diameter in the sub-hepatic area, which was larger than that of 2 days previously, dilatation of biliary tract and gall bladder, and ascites (Fig.). Perforation of the biliary tract associated with ERCP was suspected. His vital signs were unstable and he was managed in the intensive care unit. Emergency surgery was planned to drain the fluid. On the preoperative coagulation screening, prothrombin time-international normalized ratio (PT-INR) was prolonged to 9.34, which was likely because of a biliary obstruction and DOAC use. On laparotomy, biliary ascites encapsulated by the liver, stomach, and omentum was observed, which suggested a bile leak. After aspirating the biliary ascites, cholecystectomy was performed and a plastic tube (C-tube) was inserted into the CBD via the cystic duct stump to search for the site of the bile leak. Intraoperative cholangiography with X-ray contrast medium (meglumine sodium amidotrizoate) revealed no outflow into the duodenum, which suggested an obstruction of the papilla of Vater (Fig.). Dye contrast with indigo carmine revealed a leak at the periphery of the lateral hepatic segment, which suggested a bile duct rupture that was caused by the increased intra-biliary pressure. Despite administering blood transfusions (fresh frozen plasma [FFP], 14 units; red blood cells [RBC], 6 units) and vitamin K, bleeding mainly from the gallbladder bed was difficult to control and blood pressure could not be maintained. Surgical gauze was packed at the bleeding site and in the abdominal cavity to terminate the operation. After administration of FFP, RBC, and vitamin K,

PT-INR improved to 1.65 the next day. Reoperation was then performed. With dye injection through the C-tube, the site of the bile leak was identified at the periphery of the lateral hepatic segment, which was thought to be the LTL, and partial hepatic resection by ligation was performed with adequate abdominal irrigation and insertion of drains into the abdominal cavity (Fig.). Bile excretion (approximately 150 mL) was observed daily from the C-tube, but jaundice did not improve (Fig.). On postoperative day 5, ERCP was performed again. Although the site of EST looked normal, ERBD was placed again (Fig.). Jaundice then gradually improved (Fig.). Pathological findings in the surgical specimen included bile ducts, arteries, and veins in a fibrous connective tissue with neutrophil infiltration and fibrin deposition in the surrounding area. The bile duct wall showed a partial defect, which was assumed to be a perforation site. There was no significant finding that suggested bile duct fragility. Liver tissue was not detected in the specimen. Based on the pathological findings, the remnant bile ducts in the appendix fibrosa hepatis (AFH) perforated spontaneously because of increased intraductal pressure in the bile duct (Fig.). During his postoperative course, the patient had respiratory distress syndrome and accompanying multiple organ failure. He also had liver dysfunction, recurrent jaundice, and renal dysfunction that required continuous hemodialysis. He died on postoperative day 48.'

Case 3

Case 3 is adapted from 'patient 3' in https://www.ncbi.nlm.nih.gov/pmc/articles/PMC8202400, which is patient '8202400-3' in our dataset.

Input summary: 'A 57-year-old man with stage IIIC melanoma was treated with vemurafenib for 8 years with complete response until the disease progressed with brain, lung and liver metastases. After stereotactic radiotherapy, he received nivolumab but progressed again in 2 months later in lung and liver metastases, showing hepatic failure and obstructive jaundice, with LDH value was superior to two-times.'

Similar patients retrieved from PMC-Patients using BM25:

1. *Article PMID*: '33126538';

   Article title: 'Trametinib Induces the Stabilization of a Dual GNAQ p.Gly48Leu- and FGFR4 p.Cys172Gly-Mutated Uveal Melanoma. The Role of Molecular Modelling in Personalized Oncology';

   patient_uid: '7662249-1';

   Patient note: 'The patient, a 57-year-old female Caucasian, was diagnosed with uveal melanoma in the posterior and superior quadrants of the choroid of the right eye in September 2014. Following the eighth edition of the American Joint Committee on Cancer (AJCC), the tumor was classified as T4cN0M0 and therefore stage IIIB. The size of the tumor was 23.2 \u00d7 21.7 mm, with a thickness of 9.6 mm. There was no ciliary involvement and an extrascleral extension of 4.9 \u00d7 4.6

mm, and a thickness of 1 mm, was detected. The patient received a local therapy by proton beam radiotherapy. In June 2015, three liver metastases were detected by a control magnetic resonance imaging (MRI), and treated by local thermal-ablation. The patient progressed in May 2017 with lung, subcutaneous, and liver metastases. Systemic immunotherapy with the combination of ipilimumab and nivolumab was started. After three cycles, the patient experienced autoimmune thyroiditis, and the treatment was stopped. The thyroiditis resolved within a month. The patient then received one additional cycle of nivolumab, complicated by steroid-resistant autoimmune hepatitis, and the immunotherapy was definitely discontinued. In October 2017, the patient presented with the progression of subcutaneous nodular lesions, while lung and liver lesions remained stable. The liver lesions were again treated with thermal-ablation combined with hepatic radio-embolization. In April 2018, systemic progression and five new brain metastases were detected. Brain metastases were treated with stereotactic radiosurgery (SRS). Next, an in-house developed NGS, including the complete exons of 394 cancer-associated genes, was requested on one of the subcutaneous metastases to identify actionable genomic alterations. Three potentially pathological mutations were detected: BAP1 c.68-4_84delinsGA (p.?), FGFR4 c.514T > G (p.Cys172Gly) and GNAQ c.142_143delinsTT (p. Gly48Leu), with allelic frequencies of 82%, 47% and 41%, respectively (). Based on the regions covered by our panel, we determined a relatively low tumor mutation burden (TMB) (2 non-synonymous somatic mutations/Mb), which is typical of uveal melanoma and in part could explain the absence of response to immune therapy [,]. Immunohistochemistry analysis showed a PD-L1-negative tumor (Tumor Proportion Score, TPS = 0%). The functional significance of the GNAQ mutation was described as uncertain in publicly available variant databases.\nConsequently, molecular modelling was requested, which predicted a potential activating role of the GNAQ mutation. The FGFR4 mutation was considered not targetable by specific FGFR1-3 inhibitors, such as erdafitinib or by non-specific kinase inhibitors such as sorafenib. In addition, we considered the GNAQ mutation downstream of FGFR activity and hence expected an effect of MEK inhibition also on FGFR4 signalling. Based on the results of NGS and molecular modelling, the MTB recommended MEK inhibitor therapy with trametinib at 2 mg/day, every day, which was started in May 2018. MEK inhibitors have long been tested in uveal melanoma due to the activation of the MAPK pathway by GNAQ. So far, only a limited efficacy of MEK inhibitors was detected, in immune therapy naive patients with classical GNAQ mutations [,,]. Despite the absence of strong clinical evidence for MEK inhibitors in uveal melanoma and in the absence of other alternative therapies, we proposed trametinib. An additional reason for proposing a MEK inhibitor in our immune therapy exposed patient is that in patients with NRAS mutant melanoma, MEK inhibitors showed a better response rate and progression-free survival (PFS) in immune therapy-exposed patients than in immune therapy-na\u00efve patients in the NRAS-mutant melanoma (NEMO) trial []. The reason for this apparent difference remains unclear. After two months of treatment, we detected a response (). The patient experienced a grade III mucositis in August

2018, and the treatment had to be suspended for one month. During this time, we detected one new brain lesion, which was treated by Stereotactic Radiosurgery (SRS) (24Gy). In order to avoid further mucositis, the dose of trametinib was halved (1 mg/day). A repeat MRI showed two more new brain metastases, which, again, were successfully targeted with SRS (24Gy). After the initial response, the patient maintained a stable disease but eventually progressed after ten months of treatment, and trametinib was discontinued. Seventy-four months after the primary diagnosis and 32 months after the presentation of the case in the TBM, the patient remains alive.'

2. *Article PMID*: '32670650';

Article title: 'Response to Ipilimumab/Nivolumab Rechallenge and BRAF Inhibitor/MEK Inhibitor Rechallenge in a Patient with Advanced Metastatic Melanoma Previously Treated with BRAF Targeted Therapy and Immunotherapy';

patient_uid: '7334770-1';

Patient note: 'We present the case of a 62-year-old male with advanced melanoma who followed an unconventional treatment path (see). The patient was diagnosed with stage III BRAFV600E LDH normal melanoma of the right chest in June of 2011. Upon diagnosis, 4 axillary lymph nodes were positive on axillary lymph node dissection. The patient initially decided to forego treatment; however, imaging 9 months later showed numerous subcutaneous and pulmonary metastases. The patient began treatment with BRAF inhibitor vemurafenib in April of 2012. Subsequent serial PET/CT scans two months later indicated mixed response to therapy, with resolution of pulmonary and numerous subcutaneous nodules with the development of multiple new nodal and subcutaneous lesions. Throughout the following two years of vemurafenib therapy, this pattern of new and resolving nodal and subcutaneous metastases continued. Due to an overall decrease in disease burden and patient preference, treatment was continued. In September of 2014, vemurafenib was discontinued due to CT and MRI indicating significant metastases in the brain and bones. The patient completed a course of radiotherapy to L4 and L5 lesions as well as radiosurgical and radiotherapy treatment for the brain metastasis with good response. He was then started on ipilimumab for systemic therapy. After 4 cycles, CT and MRI demonstrated stable disease. Immunotherapy was held due to a period of colitis, but on resolution, a maintenance dose of ipilimumab was given. However, due to significant cutaneous metastasis, ipilimumab was discontinued in late May of 2015, and the decision was made to proceed with wide excision of the subcutaneous masses and hold systemic therapy with reimaging in 6 weeks. At that time, there were no new cutaneous metastases and intracranial/osseous disease was stable. Follow-up imaging was scheduled for another 6 weeks, which again showed stable disease even with continued hold of systemic therapy. At this point, follow-up MRI brain and CT abdomen/pelvis were scheduled for 3 months later.\nFollow-up imaging was not completed until January of 2016 but showed stable intracranial and osseous disease with multiple new cutaneous metastases. The lesions were excised, and systemic treatment options were discussed at a tumor

board. The patient was lost to follow-up until mid-June 2016, where repeat CT indicated significant progression of cutaneous metastasis. Given continued cutaneous progression, single-agent pembrolizumab was started in June of 2016. The patient had an initial mixed response to pembrolizumab with overall stable disease and remained on the therapy for 9 months. Subsequent PET/CT scan in March of 2017 showed an increased number of pulmonary nodules and approximately 7 new subcutaneous lesions on the patient's legs bilaterally, and pembrolizumab was discontinued.\nAfter discussion, ipilimumab/nivolumab combination therapy was started in April of 2017. After 4 cycles, a PET/CT demonstrated stable disease with the exception of a new subcutaneous metastasis on the ankle. Given the history of extensive treatment and lack of significant progression, single-agent nivolumab was continued and the lesion was treated palliatively with radiation therapy. Imaging in October of 2017 indicated a mixed response, with stable visceral disease but progression in the form of multiple new subcutaneous metastases in the back and right thigh. Lack of open slots in appropriate clinical trials led to continued therapy with nivolumab beyond progression. However, after imaging in December of 2017 showed continued subcutaneous progression, single-agent nivolumab was discontinued. The patient was then started on a clinical trial with intralesional SD-101+systemic pembrolizumab from January 2018 to March 2018 until progression.\nSince it had been over 3 years since the patient trialed BRAF inhibitor therapy and the patient had never been treated with BRAF inhibitor/MEK inhibitor combination therapy, dabrafenib/trametinib was initiated. The patient first began dabrafenib and trametinib in March of 2018 and had an impressive response with substantial shrinkage of subcutaneous lesions within a few days. In July of 2018, PET/CT showed near-complete response to treatment, with resolution of the pulmonary and subcutaneous nodules (). Several areas of hypermetabolic subcutaneous infiltration were seen throughout the body consistent with an inflammatory dermatologic reaction. Response persisted until unfortunately in November of 2018, CT showed evidence of progression with new nodal and soft tissue lesions as well as a single hepatic lesion concerning for metastasis. Dabrafenib/trametinib combination therapy was discontinued.\nGiven the fact that the patient progressed on all standard lines of treatment and there was no eligible clinical trial available at the time, we decided to proceed with encorafenib/binimetinib combination therapy. The patient was on encorafenib/binimetinib from December 2018 through April 2019 with partial response, until unfortunately imaging indicated progressive disease with new left external iliac nodal metastasis and soft tissue deposits.\nAs the patient had exhausted and progressed on all standard lines of treatment and no clinical trial was available for him at the time, we discussed potential treatment with nab-paclitaxel chemotherapy or retrial of ipilimumab/nivolumab immunotherapy. The patient had shown response to ipilimumab/nivolumab therapy in the past, with progression in the maintenance phase. This context was paired with data from studies suggesting the potential for immunotherapy after BRAF inhibition owing to favorable modulation of tumor microenvironment [,]. The patient decided to proceed with combination

ipilimumab/nivolumab immunotherapy and began treatment in April of 2019. PET/CT in late-July 2019 showed mixed response to therapy, with a significant decrease in nodal and subcutaneous FDG avidity with a stable small left hepatic lobe lesion that was previously noted in November 2018 (). After 5 treatment cycles, CT imaging in September 2019 showed a decrease in nodal metastasis size, a stable hepatic lesion, and no evidence of pulmonary disease. CT imaging completed 11/2019 showed no change in the nodal or hepatic lesion, indicating stable disease.'

3. *Article PMID*: '30305172';

Article title: 'Immune-related adverse events with immune checkpoint inhibitors affecting the skeleton: a seminal case series';

patient_uid: '6180387-2';

Patient note: 'Patient 2 is a 52-year old male who was originally diagnosed in 2011 with a localized BRAF V600E- melanoma of the left flank, and was treated with wide local excision (Breslow thickness: 2.8 mm) and adjuvant interferon alpha. Unfortunately he developed recurrent disease in 2014 with new lung metastases, and was treated with high-dose interleukin-2 (IL-2). His disease progressed through this therapy, with the development of new osseous metastases in the axial and appendicular skeleton. He was subsequently treated with nivolumab in combination with IL-21 on a prospective clinical trial for 8 cycles of combination therapy, followed by nivolumab monotherapy. He went on to have a near complete response to ICI therapy by RECIST 1.1, with his known osseous metastases in the ribs, pelvis, femur, humerus and vertebral bodies L3 / L4 showing sclerotic change consistent with treatment response. No skeletal radiation was administered. Given his near complete response, ICI therapy was discontinued. Seven months following the cessation of therapy, the patient developed new brain metastases, pulmonary metastases, and a paraspinal metastasis at S3. The patient was treated with stereotactic radiosurgery (SRS) of the paraspinal mass and brain and was initiated on second-line dabrafenib and trametinib. After 8-months, there was an interval increase in size of the S3 paraspinal mass, and nivolumab was re-challenged. The patient went on to receive 9-months of additional ICI therapy at which time the first vertebral fracture \u2013 not associated with a metastatic lesion \u2013 was detected. The patient\u2019s cancer was deemed to be stable is at all known sites of disease at that time. Specifically, on surveillance CT imaging, compression deformities of T2\u20135 were identified with new compression fractures noted at T6\u201312 and L1 at the time of clinic visit and vertebral fracture assessment. There was only one sclerotic lesion in the thoracic spine (T7) identified as a metastatic focus of disease; the remaining compression fractures developed in the absence of skeletal metastases. The patient\u2019s biochemical evaluation was unremarkable. Bone density testing showed only osteopenia at the femoral neck. For treatment, he received denosumab injections every 6-months. At that time, he commenced third-line ipilimumab /nivolumab combination therapy. While the patient did not suffer additional fractures, his melanoma progressed, and he passed away 7-years after initial diagnosis.'

4. *Article PMID*: ‘28807048’;

Article title: ‘Response to single agent PD-1 inhibitor after progression on previous PD-1/PD-L1 inhibitors: a case series’;

patient_uid: ‘5557522-3’;

Patient note: ‘A 78-year-old gentleman was diagnosed with stage IVM1c BRAFV600mutant cutaneous melanoma with metastases to the kidney, adrenal, and lymph node. The patient began treatment with a vemurafenib, a BRAF inhibitor, but discontinued after two months for progressive disease. He then progressed through treatment with cytotoxic chemotherapy, ipilimumab, and a combination of anti-BRAF/MEK (dabrafenib plus trametinib) combination therapy. He later received therapy with anti-PD-1 pembrolizumab for 5 months before being discontinued for treatment-related insulin-dependent diabetes mellitus, chronic pruritus, and joint pain. The patient had a complete response on treatment. Approximately 20 months after the patient\u2019s last dose of pembrolizumab, he progressed and initiated nivolumab every 2 weeks for 3 doses. Although the patient continued on insulin for treatment of diabetes mellitus throughout his time on nivolumab, neither the pruritus nor the joint pain recurred after treatment with nivolumab. Treatment was discontinued after 7 weeks due to disease progression in the bilateral adrenal lesions, lymph nodes, and a new lesion in the liver.’

5. *Article PMID*: ‘27895919’;

Article title: ‘Recurrent pleural effusions and cardiac tamponade as possible manifestations of pseudoprogression associated with nivolumab therapy\u2013 a report of two cases’;

patient_uid: ‘5109681-1’;

Patient note: ‘A 46-year old male non-smoker presented in December of 2007 with right supraclavicular lymphadenopathy. An excision biopsy of the lymph node found small cell lung cancer. A combined PET-CT (Positron Emission Tomography-Computed Tomography) scan showed a 5 cm right hilar mass and right paratracheal lymphadenopathy. He had no disease elsewhere. An MRI (Magnetic Resonance Imaging) of the brain was negative for metastatic disease.\nThe patient was referred to our institution for treatment in January 2008. He had a low-grade disease and favorable response to various therapies, and a prolonged disease course as delineated in Fig. . He was initially treated with cisplatin and etoposide and concurrent radiation therapy. He achieved complete response after 6 cycles of chemotherapy, and subsequently underwent prophylactic cranial irradiation. He was monitored clinically and by imaging every 3 months. In May 2009 the disease relapsed with left supraclavicular lymphadenopathy, confirmed by excision biopsy. He underwent radiation therapy with concurrent cisplatin and etoposide for 2 cycles followed by 4 cycles of oral topotecan. He had complete response again that lasted for a year. He had 2 further courses with platinum and etoposide due to relapsed disease in 2010 and 2011. Due to relapse in his right hilar and paratracheal lymph nodes, he was treated on a phase I trial of an Aurora kinase inhibitor in 2012 with a complete response that lasted about 18 months. Then he progressed to develop

aortocaval lymphadenopathy. He again received carboplatin/etoposide with initial response but developed a malignant pleural effusion and worsening retroperitoneal adenopathy after receiving 5 cycles. Over the next 18 months, he received several agents (topotecan, everolimus, temozolamide, docetaxel and sunitinib) with only stable disease as best response. His disease progressed to involve several organs including brain, spinal cord, liver, pancreas, adrenals, bone and pleural, pericardial and peritoneal spaces. During this time he underwent several palliative procedures including two resections of intramedullary metastases, multiple sessions of stereotactic brain radiation therapy, and ureteral stents to relieve obstruction.\nHe was then started on nivolumab (3 mg/kg every 2 weeks) in August 2015 based on preliminary results from a phase I/II study []. He had a transient increase in right paratracheal tumor size causing Superior Vena Cava (SVC) syndrome that required stenting of the SVC. He also developed rapidly accumulating bilateral pleural effusions requiring a total of six thoracenteses over the next 8 weeks. He further experienced pericardial effusion with tamponade requiring pericardiocentesis on week 9 after initiation of nivolumab (Fig.). Cytologies from both pleural and pericardial fluid were positive for malignancy. Pericardial fluid cytology showed 5 % lymphocytes. The treatment was continued every 2 weeks without any break. He had evidence of partial response at 8 weeks of therapy and near complete response at 16 weeks of therapy in December 2015 (Fig.). He did not require any further pleural or pericardial drainage after the first 2 months of therapy, and he continues to remain on treatment to date.’
